# Quality of Care Perceived by Older Patients and Caregivers in Integrated Care Pathways With Interviewing Assistance From a Social Robot: Noninferiority Randomized Controlled Trial

**DOI:** 10.2196/18787

**Published:** 2020-09-09

**Authors:** Roel Boumans, Fokke van Meulen, William van Aalst, Joyce Albers, Marèse Janssen, Marieke Peters-Kop, Getty Huisman - de Waal, Alexandra van de Poll, Koen Hindriks, Mark Neerincx, Marcel Olde Rikkert

**Affiliations:** 1 Geriatric Department Radboud University Medical Center Nijmegen Netherlands; 2 Department of Electrical Engineering Eindhoven University of Technology Eindhoven Netherlands; 3 Center for Sleep Medicine Kempenhaege Foundation Heeze Netherlands; 4 Geriatric Department Canisius Wilhelmina Ziekenhuis Nijmegen Netherlands; 5 IQ healthcare Radboud University Medical Center Nijmegen Netherlands; 6 Social AI Group Vrije Universiteit Amsterdam Netherlands; 7 Faculty of Electrical Engineering, Mathematics and Computer Science Delft University of Technology Delft Netherlands

**Keywords:** integrated care pathway, social robot, quality of care, noninferiority randomized controlled trial

## Abstract

**Background:**

Society is facing a global shortage of 17 million health care workers, along with increasing health care demands from a growing number of older adults. Social robots are being considered as solutions to part of this problem.

**Objective:**

Our objective is to evaluate the quality of care perceived by patients and caregivers for an integrated care pathway in an outpatient clinic using a social robot for patient-reported outcome measure (PROM) interviews versus the currently used professional interviews.

**Methods:**

A multicenter, two-parallel-group, nonblinded, randomized controlled trial was used to test for noninferiority of the quality of care delivered through robot-assisted care. The randomization was performed using a computer-generated table. The setting consisted of two outpatient clinics, and the study took place from July to December 2019. Of 419 patients who visited the participating outpatient clinics, 110 older patients met the criteria for recruitment. Inclusion criteria were the ability to speak and read Dutch and being assisted by a participating health care professional. Exclusion criteria were serious hearing or vision problems, serious cognitive problems, and paranoia or similar psychiatric problems. The intervention consisted of a social robot conducting a 36-item PROM. As the main outcome measure, the customized Consumer Quality Index (CQI) was used, as reported by patients and caregivers for the outpatient pathway of care.

**Results:**

In total, 75 intermediately frail older patients were included in the study, randomly assigned to the intervention and control groups, and processed: 36 female (48%) and 39 male (52%); mean age 77.4 years (SD 7.3), range 60-91 years. There was no significant difference in the total patient CQI scores between the patients included in the robot-assisted care pathway (mean 9.27, SD 0.65, n=37) and those in the control group (mean 9.00, SD 0.70, n=38): *P*=.08, 95% CI –0.04 to 0.58. There was no significant difference in the total CQI scores between caregivers in the intervention group (mean 9.21, SD 0.76, n=30) and those in the control group (mean 9.09, SD 0.60, n=35): *P*=.47, 95% CI –0.21 to 0.46. No harm or unintended effects occurred.

**Conclusions:**

Geriatric patients and their informal caregivers valued robot-assisted and nonrobot-assisted care pathways equally.

**Trial Registration:**

ClinicalTrials.gov NCT03857789; https://clinicaltrials.gov/ct2/show/NCT03857789

## Introduction

In 2019, society was facing a global shortage of 17 million health care workers [1], along with increasing health care demands from a growing number of older adults [2]. Social robots are being considered as solutions to part of this problem [3,4]. For example, social robots—humanoid robots that are capable of social interaction with humans [5]—might be able to support professionals in hospital-implemented integrated care pathways [6].

Such pathways are already being used to optimize workforce use and cost-effectiveness by delivering health care for a well-defined group of patients during a well-defined period [7]. The overall aim of a care pathway is to enhance the quality of care by improving patient outcomes, promoting patient safety, increasing patient satisfaction, and optimizing the use of resources [7]. Pathways also make it possible to standardize certain parts of communication with patients (eg, for information on the process of care and questionnaires needed to assess outcomes) [6]. A care pathway can be visualized in the form of a time diagram (see Multimedia Appendix 1, Figure MA1-1) depicting the aims of the pathway steps and the responsible health care professionals who interact with the patient. Although all of these dialogues are important, not all may require the actual presence of health care professionals. Some could be carried out by social robots, under the supervision of health care professionals.

Many studies have been conducted on assistive robots for health care professionals [8], as well as on the cost-effectiveness of care pathways. We focus on health care robots that perform a verbal health care–related interaction with patients. For example, Di Nuovo et al used the social robot Pepper to study the assessment of cognitive skills of university personnel with the Montreal Cognitive Assessment (MoCA) [9-11]. Bandera et al designed CLARC (CLinical Assistant Robot for Comprehensive geriatric assessment), a robot designed to perform a comprehensive geriatric assessment, but have not yet published results on its interviewing performance [12-14]. Broadbent et al used a robot to provide at-home assistance to people with chronic obstructive pulmonary disease. This robot spoke but could not listen; patients entered their responses on a touch screen [15]. D’Onofrio et al describe the MARIO (Managing active and healthy Aging with use of caRing servIce rObots) robot that was designed for the practical daily living support of people with dementia in nursing homes, focusing on differences in feasibility between the United Kingdom, Ireland, and Sweden [16]. An evaluation of a social robot conducting interviews using medical questions with community-dwelling older adults has been described in Boumans et al [17]. In a crossover study, 31 participants were subjected to a question-and-answer dialogue with the robot that included personalization and affective statements. Participants scored the robot’s subjective usability, on average, as 80.1 (SD 11.6) on a scale from 0 to 100. Subsequently, they performed an ecological validation on the agreement of data collected by automated acquisition for three complete patient-reported outcome measures (PROMs), also among community-dwelling older adults. Data acquisition by a humanoid robot was compared to acquisition by a nurse in a crossover study. The conclusion was that a moderate-to-substantial agreement could be demonstrated between the frailty, well-being, and resilience scores [18]. The Lio robot (F&P Robotics) is appreciated as a support to older adults in care homes for functions such as handing over physical objects and support in performing exercises, but is not used for medical interviewing [19]. The same is true for the Care-O-bot 4 robot (Fraunhofer Institute) [20]; however, the development of this robot has been reported to be discontinued [19]. To our knowledge, however, no studies have been conducted on the quality of care, acceptance, and efficiency of social robots as an integrated part of care pathways in an outpatient clinic.

This study is, thus, the first to evaluate robot interaction with older patients within the outpatient clinic context. The older patient population was chosen, as their consultations often take more time and are more complex—due to sensory and cognitive impairments—than those for younger and less complex patient groups. Our target group thus allows substantial room for robot-assisted support.

Our hypothesis is that the quality of care perceived by patients and caregivers in a pathway that includes a social robot for a standardized part of health care professional–patient dialogue is not significantly lower than that perceived by the control group, whose pathway involves the continued presence of health care professionals; this is a noninferiority hypothesis. Perceived quality of care can be measured validly and reliably using the Consumer Quality Index (CQI) [21], which has been used to monitor the quality of outpatient clinics in all Dutch hospitals [22].

## Methods

### Study Design

The study was designed as a between-subjects, multicenter, randomized controlled trial among patients visiting the outpatient memory clinics at two teaching hospitals: Radboud university medical center and Canisius Wilhelmina Ziekenhuis. The study was conducted between July and December 2019. The care pathways of both clinics consisted of six steps: a welcome, a physical examination, an interview using a PROM and a frailty questionnaire, a discussion of the results, a discussion on any other relevant medical issues, and a farewell (see Multimedia Appendix 1). We selected a care pathway describing older patients’ repeated outpatient visits to control for safe and effective use of medications, such as cholinesterase inhibitors in patients with early-stage dementia. In the intervention pathway, the PROM and frailty questionnaires were administered by the robot, with all other actions performed by the health care professional. In the nonintervention pathway, all tasks were performed by the health care professionals. The Older Patients and Informal Caregiver Survey – Short Form (TOPICS-SF) was used as the PROM and frailty questionnaire. It consists of 36 questions on general health outcome measures: pain and discomfort, memory, activities of daily living, feelings, social activities, and current diseases [23]. The questionnaire results are used to generate a Frailty Index (FI), which is calculated as the summation of the values associated with each answer, divided by the total of answered questions. The feasibility, validity, and reliability of the instrument as a frailty questionnaire has been established in previous studies [24,25]. It has also been validated as a PROM [23]. The TOPICS-SF is currently accepted by the Dutch Geriatrics Society as a PROM for older patients throughout the Netherlands, and it is being implemented within several hospitals throughout the country [25]. The TOPICS-SF is included in Multimedia Appendix 2.

### Patient Population

Patients were recruited from the group of patients scheduled to visit the outpatient clinics of the geriatrics departments of both hospitals. These outpatient clinics subsequently welcomed a total of 419 patients during the study. Inclusion criteria were the ability to speak and read Dutch and being assisted by one of the regular staff nurses or physicians taking part in the study. Exclusion criteria were serious hearing or vision problems, serious cognitive problems, and paranoia or similar psychiatric problems, all as judged by the health care professional, as well as situations in which the patient had previously been asked to complete the TOPICS-SF. The patient population for this noninferiority trial was similar to the population that would be included in a trial for establishing the efficacy of social robots. Patients were selected by their responsible health care professionals, based on the inclusion criteria, upon reviewing the patient visits scheduled in the electronic health record (EHR) system. Patients were screened for exclusion criteria and consent was requested, all according to a standardized script.

### Public and Patient Involvement

Patients were involved in the study as subjects; the public was involved in the study through the patients’ accompanying informal caregivers and patient organization representatives. The study hypothesis explicitly refers to the measurement of patients’ opinions by using the CQI. The public has also been involved in the study design through the preceding studies among community-dwelling older adults [26,27] and through advice given from patient organization representatives during pilot tests. The minimization of the burden on, and time required of, the patients was an important criterion in the study design.

### Randomization

Patients were randomized using a computer-generated list and assigned to either the intervention or the control group, in sequence of admission. The nature of the intervention prevented the blinding of group allocation, and data acquisition could not be blinded from the patient perspective, given that the data were self-reported.

### Study Procedure

The health care professional guided the patient from the waiting room to an examination room, where the robot was or was not present, depending on randomization.

**Figure 1:**
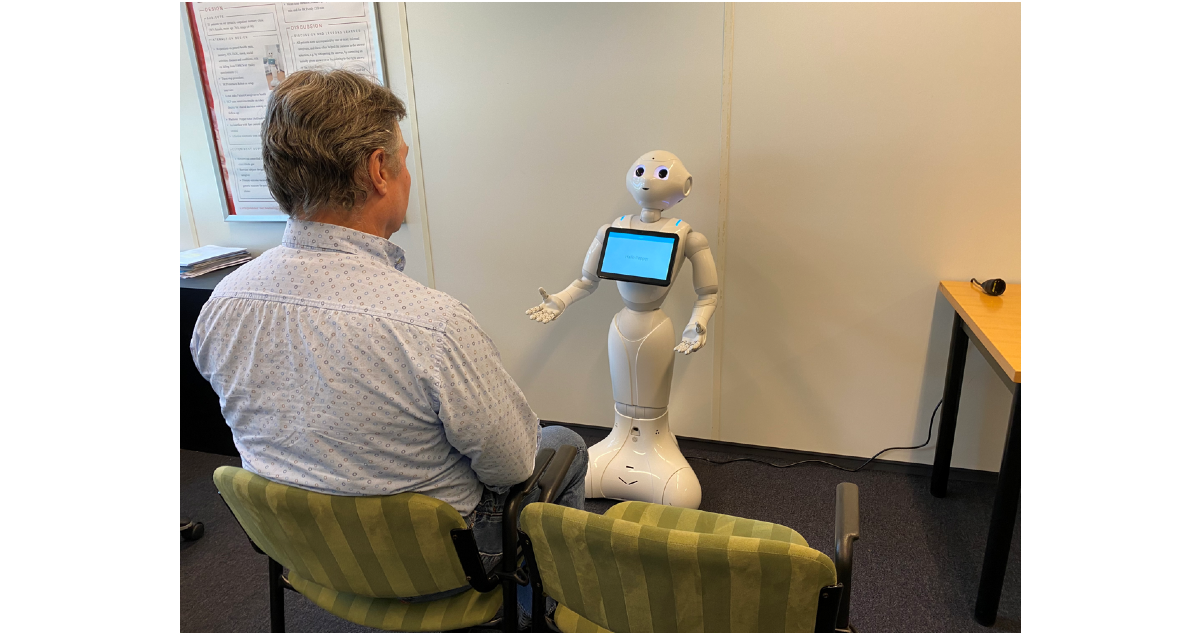
The robot-patient interview setup. The patient sits on the right (not shown) and the informal caregiver sits on the left (person shown was not part of the study population). The distance between the patient and the robot is 90 cm.

In the intervention pathway, the health care professional started the interview with several open-ended questions on the patient’s general health status. This was followed by the introduction of the robot, which subsequently conducted the TOPICS-SF interview. Upon completing the interview, the robot generated a report of the PROM and FI results, including the activities of daily living and the instrumental activities of daily living scores. This report was the input for subsequent interactions between the patient and the health care professional within the context of shared decision making [28]. The robot-patient interaction is detailed in Multimedia Appendix 3. The interview setup is shown in Figure 1. A video of the interaction is shown in Multimedia Appendix 4.

In the control group, following the initial general talk, the health care professional started the structured TOPICS-SF. The results were discussed with the patient, and the other parts of the medical examination and management plan were carried out.

If needed, these steps were followed by other medical procedures that had been scheduled for the patient’s care pathway (eg, blood samples, electrocardiogram, and MoCA) [8]. In both scenarios, if there were no more medical issues to handle, a research assistant asked both the patient and the caregiver to complete the CQI questionnaire. After the CQI questionnaire was checked for completeness, the health care professional completed the visit and said farewell.

### Human-Robot Interaction Design

The social robot used in this study was a Pepper robot, version 1.8a, using the NAOqi operating system, version 3.9 (SoftBank Robotics) [10]. The robot software necessary for the intervention was designed and programmed using Android Studio, version 3.1 (Google Inc), and Java, version 8 (Oracle Corp). The software managing the dialogue included rules for introducing question groups, for providing variability in how similar questions were asked, and for generating affective and connecting statements. Answers were stored directly in the hospital’s EHR system. Ethical design considerations were taken into account by incorporating the fundamentals of care [29] into the communication design. For example, for each question, the default answer set was divided into two groups: (1) answers indicating serious conditions, which could possibly invoke empathy on the part of a health care professional, and (2) answers indicating minor conditions, which would not require separate discussion. The robot looked mostly at the patient and sometimes at the caregiver, in order to create engagement with both. The robot’s tablet display was used to show each question and the associated answer options. The layout of the interaction design was based on guidelines for older adults [26]. After hearing the patient’s answer, the robot repeated it and showed it on the display, then proceeded to the next question. More details are provided in Multimedia Appendix 3.

### Training Health Care Professionals

For this experiment, secretarial staff members were trained in using a telephone script and a list of answers to frequently asked questions about the robot. These answers were used in the event that patients or caregivers called with questions. Health care professionals were trained in how to start the robot, interact with the EHR system through the robot, initiate the questioning, and use the questionnaire report on the tablet.

### Primary Outcome

The most relevant part of the validated, general medical CQI questionnaire for outpatient clinics was selected as the primary outcome measure [27,30]. Most of the list items were not applicable to our study and in the attempt to minimize the burden to the patients, the 10 most relevant questions were selected in advance (see Multimedia Appendix 5, Table MA5-1). This selection was done in line with recommendations for shortening the CQI questionnaires [22,31]. Furthermore, the subscales regarding the clinic and the treatment by the health care professional showed Cronbach α values of .845 and .880, respectively, thus indicating a high degree of correlation in the subscales [32]. Therefore, we considered our selection of relevant questions as allowed. Answers were evaluated for the scale as a whole, for the two subscales (ie, regarding the clinic and regarding the robot-supported health care professional), and individually.

**Figure 2:**
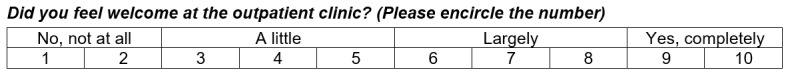
An example of one of the 10 Consumer Quality Index (CQI) questions; this is presented in its 10-point scale version.

Answers to the CQI questions are generally scored categorically, including *no, not at all*; *a little*; *largely*; and *yes, completely*. The granularity of this scale is small, however, and pilot evaluations revealed ceiling effects and skewed distributions. The patients were, therefore, asked to assign scores on a scale from 1 to 10, with references to these categories (see Figure 2).

The opinion of the informal caregiver accompanying the patient was also recorded using the same questions, albeit reformulated for the informal caregiver’s perspective. The answers to each CQI question were averaged across all patients and caregivers in each group. The primary outcome was then calculated as the mean sum of the individual question outcomes. The same method was used for the two aforementioned subscales.

### Secondary Outcomes

The time duration of the TOPICS-SF interview was registered as a secondary outcome by observers who witnessed each interview. These observers further used an observation form to record, for each question, the extent to which the patient and caregiver exchanged information on the TOPICS-SF answers (see Multimedia Appendix 6, Figure MA6-1). Other potentially relevant events were also recorded (eg, patient remarks on the interaction). The observers were instructed not to intervene at all. Given that such self-recording of secondary outcomes could not be blinded, observation bias was limited by using alternating trained observers. The general medical situation of the patient group was categorized according to the mean FI as follows: *robust* (FI ≤ 0.095), *prefrail* (0.095 < FI < 0.20), and *frail* (FI ≥ 0.20) [33]. The total number of reported comorbidities per patient was calculated, resulting in a value between 0 and 18.

In the intervention group, four questions based on the Almere model [34] were asked to evaluate the usability of the robot (see Multimedia Appendix 7, Table MA7-1). This made it possible to compare these results to our previous work [17,18]. To limit patient burden, survey questions were restricted to three variables: *perceived ease of use* (two items), *perceived enjoyment*, and *trust* [34].

### Sample Size Calculation

In our two previous robot studies, which were conducted with 30 and 40 community-dwelling older volunteers, respectively, we found hardly any difference between the answers given to the robot and those given to the health care professional [17,18]. In this study, therefore, we focused on the quality of care perceived by patients and caregivers, hypothesizing that the robot interview would also not be valued less by the intervention group. For this reason, a noninferiority, sample size calculation was applied, specifying that the mean CQI of the intervention group should not be lower than the mean CQI of the control group minus 1.0, with a standard deviation of 1.5, α=.05, and power=1–β=.90 [35]. The difference value of 1.0 is based on the guideline proposed by Ringash et al, which defines 10% of the PROM scale range as a meaningful difference [36]. This calculation resulted in a sample size of 39 patients per group (78 in total).

### Statistical Analysis

Data were stored in Castor, a cloud-based medical data management system (Castor EDC). Intention-to-treat analysis was performed using SPSS Statistics for Windows, version 25.0 (IBM Corp), and Microsoft Excel (Office 365, Microsoft). Because not all data were reported by patients or caregivers, the number of patients to which variables relate are reported separately. Missing values were not considered random and, thus, not imputed. Normally distributed values are presented as means, with standard deviations in parentheses. Because the target sample size was larger than 25, we applied the central limit theorem and assumed normality on the part of the summed score for the CQI questionnaire. Groups were compared using independent-samples *t* tests and, in case of nonnormality, the Mann-Whitney U test. For significant effects or effect trends, effect sizes were calculated as Cohen *d*.

### Ethical Considerations

The study was conducted according to the principles of the Declaration of Helsinki (2013), in accordance with the Medical Research Involving Human Subjects Act (*Wet medisch-wetenschappelijk onderzoek met mensen* [WMO] in Dutch) and the CONSORT (Consolidated Standards of Reporting Trials) guidelines for randomized controlled trials, including the extension for noninferiority trials [37]. The study protocol was approved by the Institutional Review Board from each hospital. All patients granted written informed consent. Caregivers had the option to grant consent on behalf of their relatives, but this situation did not occur. This trial was registered at ClinicalTrials.gov (NCT03857789).

## Results

### Patient Population

**Figure 3:**
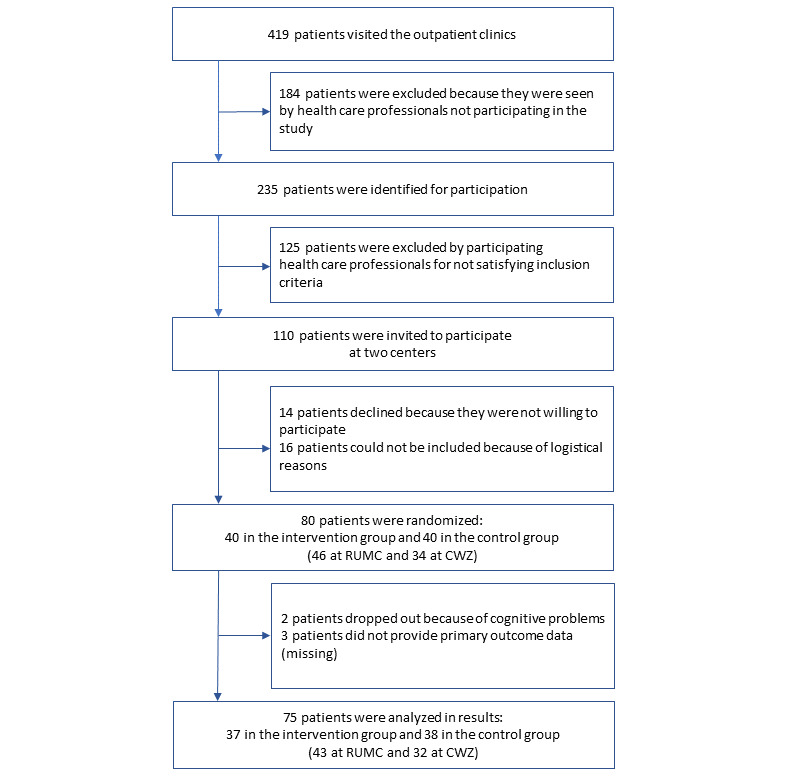
Patient flowchart. CWZ: Canisius Wilhelmina Ziekenhuis; RUMC: Radboud university medical center.

The patient flowchart is provided in Figure 3. Recruitment was stopped upon reaching 80 included patients. However, 2 patients dropped out during the experiment after randomization: 1 patient turned out to have cognitive problems that made it impossible to complete the robot interaction, and 1 patient chose to discontinue the interview with the robot after nine questions because “she did not like the robot.” Another 3 patients were lost to follow-up because of the unavailability of their CQI ratings. Therefore, the dataset used consisted of 75 patients: 36 female (48%) and 39 male (52%); mean age 77.4 years (SD 7.3), range 60-91 years. Of the 75 patients, 37 were in the intervention group (49%) and 38 were in the control group (51%).

All 75 patients were accompanied by an informal caregiver: 34 were partners of a patient (45%), 23 were children of a patient (31%), and 1 was a friend of a patient (1%); 8 informal caregivers had other affiliations (11%) and 9 did not disclose their relationship to the patient (12%).

None of the 14 patients (see Figure 3) who declined the invitation due to unwillingness to participate mentioned the robot as the reason (14/75, 19%).

The consultations were conducted by 13 different health care professionals. The patient-robot interactions were observed by 11 different trained observers. No important incidents of harm or unintended effects were observed or reported.

The FI for the group as a whole ranged from 0.07 to 0.68 (mean 0.26, SD 0.15). The mean FI for the control group (mean 0.26, SD 0.15) and the intervention group (mean 0.25, SD 0.15) were similar (*P*=.99). Out of 75 patients, 4 (5%) patients could be categorized as robust, 30 (40%) as prefrail, and 36 (48%) as frail; in addition, 21 patients (28%) had been diagnosed with dementia. The average number of comorbidities per patient was 3.9 (SD 2.6). The main patient baseline clinical data for each group are included in Table 1; extended data are provided in Multimedia Appendix 8.

**Table 1:** Baseline characteristics of the study population (N=75).

Characteristic				Intervention group (n=37)		Control group (n=38)
Sex (female), n (%)				16 (43)		20 (53)
Age (years), mean (SD)				78.1 (7.0)		76.7 (7.7)
Self-indicated quality-of-life score (0-10), mean (SD)				7.5 (1.9)		7.1 (1.6)
Frailty Index (0-1), mean (SD)				0.25 (0.15)		0.26 (0.15)
**Frailty value, n (%)**						
	Robust		3 (8)		1 (3)	
	Prefrail		13 (35)		17 (45)	
	Frail		21 (57)		15 (39)	
**Comorbidities, n (%)**						
	Memory complaints		19 (51)		26 (68)	
	**Pain**					
		None		11 (30)		14 (37)
		A little		12 (32)		9 (24)
		Moderate		8 (22)		10 (26)
		Severe		6 (16)		3 (8)
		Extreme		0 (0)		1 (3)
	Dementia		11 (30)		10 (26)	
	Hearing problems		9 (24)		8 (21)	
	Vision problems		10 (27)		3 (8)	

### Primary Outcome

**Figure 4:**
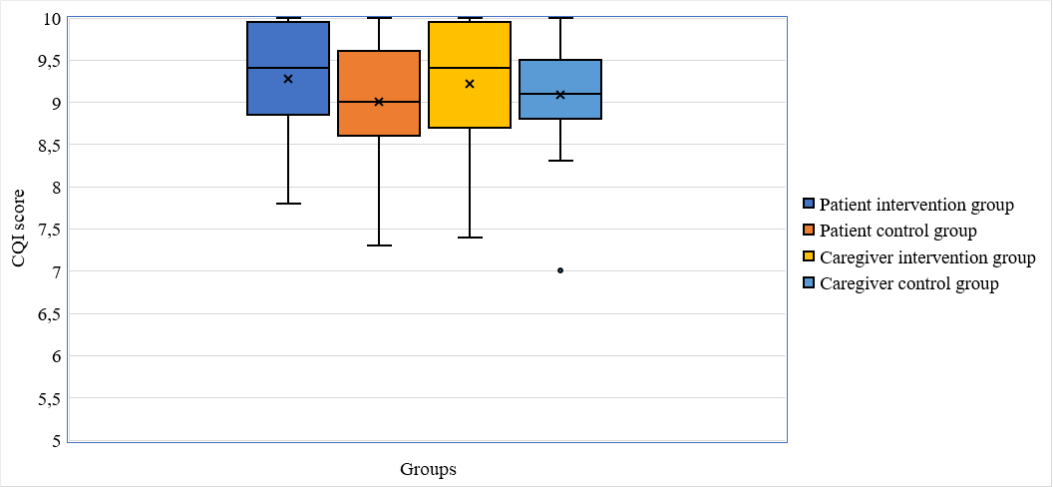
Box plots for total Consumer Quality Index (CQI) scores for patients (two box plots on the left) and caregivers (two box plots on the right).

The total CQI scores recorded for patients and caregivers are presented graphically in Figure 4. There was no significant difference in the total patient CQI scores for the intervention group (mean 9.27, SD 0.65) and the control group (mean 9.00, SD 0.70) (t_73_=1.76, *P*=.08, 95% CI –0.04 to 0.58). There was also no significant difference in the total informal caregiver CQI scores for the intervention group (mean 9.21, SD 0.76) and the control group (mean 9.09, SD 0.60) (t_63_=0.73, *P*=.47, 95% CI –0.21 to 0.46).

A *t* test on each of the 10 individual CQI questions (see Multimedia Appendix 9) revealed that patients found that health care professionals, when supported by a robot, listened better (mean 9.46, SD 0.69) than health care professionals not supported by the robot (mean 9.11, SD 0.76) (t_73_=2.104, *P*=.04, 95% CI 0.019-0.690; Cohen *d*=0.48). Patients also found that health care professionals, when supported by the robot, had more time for the patient (mean 9.54, SD 0.56) compared to those not being supported by the robot (mean 9.13, SD 0.70) (t_73_=2.784, *P*=.007, 95% CI 0.116-0.702; Cohen *d*=0.64). The other eight questions, individually, did not reveal any significant differences. A *t* test on the group of questions about the care provided by the health care professional (see Multimedia Appendix 9, questions 4-8) showed that patients found that health care professionals supported by the robot provided better care (mean 9.42, SD 0.62) than health care professionals not supported by the robot (mean 9.11, SD 0.69) (t_73_=2.086, *P*=.04, 95% CI 0.014-0.619; Cohen *d*=0.48). The patients’ answers to the group of questions about the clinic (see Multimedia Appendix 9, questions 1-3, 9, and 10) did not show significant differences. Regarding informal caregivers accompanying the patients, there were no significant differences found between health care professionals supported by a robot or not, nor between a clinic using a robot or not. The CQI scores for all questions are included in Multimedia Appendix 9, and the total CQI distributions are presented in Multimedia Appendix 10.

### Secondary Outcomes

Within the care pathways, the mean duration for completing the TOPICS-SF with the robot was 17.9 minutes (SD 5.2), as compared to 14.8 minutes (SD 10.8) for the control group. The difference was not significant: t_70_=1.60, *P*=.11, 95% CI –0.79 to 7.18. It should be noted that observations showed that health care professionals regularly skipped questions.

It was observed that patients and caregivers did not discuss the TOPICS-SF answer options any more during the interviews with the robot (mean 3.5, SD 3.8) than was the case in the control group (mean 2.9, SD 2.5): t_53_=0.58, *P*=.56, 95% CI –1.32 to 2.42. It was further observed that, at the start of the interview, patients sometimes answered before the robot was finished speaking. This well-known barge-in effect occurred despite the fact that the robot had instructed patients to wait for the blue bar to appear at the top of the tablet before speaking [38]. Most patients learned after three or four questions that it was better to wait a short while before answering, as they would otherwise have to repeat their answers. Informal caregivers occasionally helped the patients when necessary (10% of the questions); for example, because one patient spoke a local Dutch dialect that was not understood by the robot, the patient’s caregiver answered instead.

For the intervention group only, the mean scores for *perceived enjoyment*, *perceived ease of use* (2 items), and *trust* with regard to the robot interaction were recorded (see Multimedia Appendix 11). There was no significant difference in *perceived enjoyment* between patients (mean 7.81, SD 2.01) and caregivers (mean 7.56, SD 2.11): t_56_=0.47, *P*=.64, 95% CI –0.85 to 1.37. In addition, there was no significant difference in *perceived ease of use* in terms of having sufficient response time between patients (mean 8.51, SD 1.63) and caregivers (mean 8.45, SD 1.10): t_55_=0.15, *P*=.88, 95% CI –0.73 to 0.85. There was also no significant difference in *perceived ease of use* in terms of easy answering between patients (mean 8.11, SD 1.89) and caregivers (mean 7.86, SD 1.67): t_56_=0.50, *P*=.62, 95% CI –0.74 to 1.23. Trust scores were higher for patients (mean 8.42, SD 1.38) than for caregivers (mean 7.59, SD 1.76): t_55_=2.00, *P*=.05, 95% CI <–0.001 to 1.68; Cohen *d*=0.55. Of the 36 caregivers in the intervention group who answered the CQI questions, only 24 (67%) also answered the questions on robot appreciation. The caregivers who did not answer argued that it was better for the patients to answer themselves, as they had been the ones to talk to the robot.

## Discussion

### Principal Findings

To our knowledge, this study is the first to provide an assessment of patients’ perceived quality of care in integrated care pathways with and without the support of social robots. We found that the perceptions of older patients and caregivers concerning quality of care were no different from the perceptions of quality of care in a pathway in which all interactions were carried out by health care professionals. This confirmed our hypothesis of noninferiority. The opinions of the patients and caregivers concerning the robot were in line with previous findings regarding the positive appreciation results on robot interaction among community-dwelling older adults [26,27], as well as with the results reported in our exploratory study among hospitalized patients [39].

Older adult patients participating in this study who had been diagnosed with dementia (11/37, 30%) were still able to answer the questions asked by the robot. The preselection of participants by the health care professionals probably resulted in a group with mild-to-moderate cognitive problems, who were still able to communicate verbally with either the health care professional or the robot. It was also observed that patients with auditive (9/37, 24%) or visual (10/37, 27%) problems were capable of completing the interview. This indicates that the design measures taken to improve robot communication (ie, quiet environment, adjusted voice volume and speed, font size of text on the tablet, and minimalistic layout) were adequate. When they deemed it necessary, informal caregivers assisted patients; this occurred for 10% of the questions.

The observers noted that, in the control group, health care professionals regularly skipped questions from the TOPICS-SF. When asked about this, the professionals responded that they had skipped questions to which they already knew the answers or that they considered inappropriate to ask explicitly. The robot always asked all of the questions. This could be a potential advantage, as it ensures that no items will be missed inadvertently.

### Strengths and Weaknesses of the Study

The major strength of this study is that this is the first multicenter, randomized controlled trial on the acquisition of routine, collected PROM data with a social robot among older adult patients within an integrated care pathway. The noninferiority results of this trial suggest that an adequately designed social robot could be acceptable for use with older adult patients and their informal caregivers as part of an integrated care pathway, under the indirect supervision of a health care professional.

Despite this strength, this study is also subject to several limitations. First, after analysis, it turned out that the planned sample size was not met because of 2 dropouts and 3 participants with missing data, which was more than our margin of 2 patients. However, by imputing the dataset with 2 intervention group patients with scores of mean–2σ and 1 control group patient with a score of mean+2σ, which was considered as the worst case scenario, it was found that this did not affect the conclusion of a nonsignificant difference in perceived quality of the care pathway. Secondly, it was not possible to blind the assignment of patients to groups. Thirdly, the between-subjects design did not allow any comparative-accuracy analyses of the answers. In our previous study, however, the results indicated moderate-to-good agreement between scores with and without the robot [18].

### Comparison With Prior Work

The results confirm and extend those of previous studies on the use of robots outside the hospital context [10,12,15,16]. For example, Olde Keizer et al concluded that social robots could potentially monitor and train the health of frail older adults, but they also identified some critical usability challenges [40]. Furthermore, the functionality of the Lio robot (F&P Robotics), given its reported voice communication capabilities, could be extended with verbal interviewing functions as described herein.

Riek has provided a comprehensive overview of robot applications in health care with many examples of physical support [8]. This study adds to the knowledge base a multicenter, randomized controlled trial examining the verbal support option of a robot interviewing older adult patients in an outpatient clinic regarding their health and, as such, resolves part of the paucity in effective clinical trials that Riek noted [8].

### Meaning of the Study

In terms of generalizability, the patient group in this study was more frail and had more substantial multimorbidity than is the case for the general hospital population. Communication with the robot could possibly be even easier for the general hospital population. For this reason, and because the TOPICS-SF is similar to many available PROMs, it is plausible that the results can be generalized to most adults admitted to hospitals, as well as to most care pathways. The results thus suggest that robot assistance could be implemented more broadly without affecting perceived quality of care.

The observations and experiences gained in this experiment could also be translated into a number of recommendations. First, the introduction of a social robot should lead to a carefully prepared rearrangement of tasks among the health care professionals within a pathway of care. Second, for reasons of patient privacy and the intelligibility of the patient’s utterances to the robot, the robot should be a fixed element in an outpatient room. Third, participating health care professionals appreciated the direct availability of all collected data in the EHR system. Therefore, we recommend implementing real-time data export from the robot to the hospital’s EHR system for successful implementation. Fourth, technologies like these may support clinical care during pandemics, since they limit person-to-person contact and allow for social distancing.

Our findings suggest that this social-robot technology could be implemented more broadly for obtaining PROM data, as well as for other standardized parts of functional assessments and medical history taking. The assistance of social robots could, thus, potentially contribute to reducing problems related to the scarcity of health care personnel, while maintaining the quality of care, as perceived by patients and caregivers.

### Unanswered Questions and Future Research

In the course of our study, we learned that one important further step in improving robot technology involves developing the ability to speak and listen at the same time, thus allowing for *barging-in* by patients. Although such technology does exist, it was not implemented in the robot used in this study. Moreover, the quality of the robot’s speech recognition depended on its focus on the interlocutor, which was controlled by the built-in *human engagement* function. Improving the controllability of this function, in terms of both speech and body motions, would help to build rapport with users.

## Appendix

Multimedia Appendix 1 Care pathway treatment.

Multimedia Appendix 2 The Older Patients and Informal Caregiver Survey – Short Form (TOPICS-SF).

Multimedia Appendix 3 Detailed description of the robot-patient interaction.

Multimedia Appendix 4 Video of a short part of the human-robot interaction.

Multimedia Appendix 5 Consumer Quality Index (CQI) questions.

Multimedia Appendix 6 Observation form.

Multimedia Appendix 7 Robot-usability questions.

Multimedia Appendix 8 Baseline data tables.

Multimedia Appendix 9 Consumer Quality Index (CQI) scores by question.

Multimedia Appendix 10 Consumer Quality Index (CQI) distributions.

Multimedia Appendix 11 Patient and caregiver opinions regarding robot usability.

Multimedia Appendix 12 CONSORT-EHEALTH checklsit (V 1.6.1).

## Abbreviations

CLARC: CLinical Assistant Robot for Comprehensive geriatric assessment
CONSORT: Consolidated Standards of Reporting Trials
CQI: Consumer Quality Index
EHR: electronic health record
FI: Frailty Index
MARIO: Managing active and healthy Aging with use of caRing servIce rObots
MoCA: Montreal Cognitive Assessment
PROM: patient-reported outcome measure
TOPICS-SF: The Older Patients and Informal Caregiver Survey – Short Form
WMO: Medical Research Involving Human Subjects Act (Wet medisch-wetenschappelijk onderzoek met mensen in Dutch)
