# Supplementary material for: Quality of Care Perceived by Older Patients and Caregivers in Integrated Care Pathways With Interviewing Assistance From a Social Robot: Noninferiority Randomized Controlled Trial
Source: J Med Internet Res. 2020 Sep 9;22(9):e18787. doi: 10.2196/18787 (PMC7511864; doi:10.2196/18787)
Supplement: Multimedia Appendix 1 [file jmir_v22i9e18787_app1.docx]

# Multimedia Appendix 1 – Care pathway treatment

Figure MA1-1 provides a graphic representation of a sequence of activities within a care pathway for the treatment and monitoring of patients with cognitive decline. The brown-coloured blocks indicate patient-reported outcome (PROM) data-collection activities, whereas the green-coloured blocks indicate results discussion and shared decision-making. The questionnaire block could be performed by a social robot.


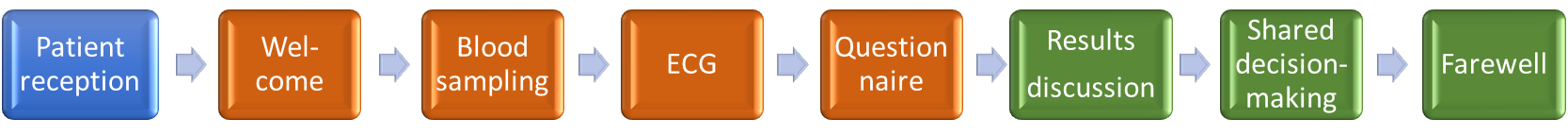


Figure MA1-1 - Care Pathway for the treatment and monitoring of patients with cognitive decline
