# Supplementary material for: Quality of Care Perceived by Older Patients and Caregivers in Integrated Care Pathways With Interviewing Assistance From a Social Robot: Noninferiority Randomized Controlled Trial
Source: J Med Internet Res. 2020 Sep 9;22(9):e18787. doi: 10.2196/18787 (PMC7511864; doi:10.2196/18787)
Supplement: Multimedia Appendix 2 [file jmir_v22i9e18787_app2.docx]

# Multimedia Appendix 2 – TOPICS SF questionnaire

The TOPICS short form includes the following questions:

Table MA2-1 - Topics short form questionnaire

| # | Question | Answer scale | Frailty Index Contribution |
| --- | --- | --- | --- |
| 1 | On a scale from 0 to 10, how do you rate your health? | 0–10 | {1, 0.9 …. 0.1, 0} |
| 2 | At this moment, do you have any pain or other complaints? | No / A little / Moderate / Serious / Extreme | {0, 0.25, 0.50, 0.75, 1} |
| 3 | Do you have any complaints with regard to your memory? | No / Yes | {0, 1} |
| 4 | Are you able to do the following fully independently:  …. get dressed and undressed? | Yes without any effort / With minor effort / With major effort / No | {0, 0.33, 0.66, 1} |
| 5 | …. get up out of a chair? | id. | id. |
| 6 | …. wash and dry your entire body? | id. | id. |
| 7 | …. walk up and down the stairs? | id. | id. |
| 8 | …. walk outside? | id. | id. |
| 9 | …. take care of your feet and toe nails? | id. | id. |
| 10 | …. perform light household tasks? | id. | id. |
| 11 | …. do your shopping? | id. | id. |
| 12 | …. take your medicines? | id. | id. |
| 13 | …. use your own or public transport? | id. | id. |
| 14 | In the past four weeks, how often: …. did you feel calm and tranquil? | Continuously / Mostly / Often / Sometimes / Rarely / Never | {0, 0.2, 0.4, 0.6, 0.8, 1} |
| 15 | …. did you feel despondent and sombre? | id. | {1, 0.8, 0.6, 0.4, 0.2, 0} |
| 16 | …. did you feel happy? | id. | {0, 0.2, 0.4, 0.6, 0.8, 1} |
| 17 | …. have your physical health or emotional problems hampered your social activities? | Continuously / Mostly / Sometimes / Rarely / Never | {0, 0.25, 0.50, 0.75, 1} |
| 18 | On a scale from 0 to 10, how do you rate your quality of life? | 0–10 | {1, 0.9 …. 0.1, 0} |
|  | Do you have or have you had one or more of the following diseases and conditions in the past 12 months? |  |  |
| 19 | Diabetes | No / Yes | {0, 1} |
| 20 | Stroke, cerebral haemorrhage (bleed in the brain), cerebral infarction (blocked blood vessel in the brain) or TIA | No / Yes | id. |
| 21 | Heart failure, myocardial infarction (heart attack) or other heart condition | No / Yes | id. |
| 22 | A type of cancer | No / Yes | id. |
| 23 | Asthma, chronic bronchitis, pulmonary emphysema or COPD | No / Yes | id. |
| 24 | Involuntary urinary loss (incontinence) | No / Yes | id. |
| 25 | Wearing of the joints (arthrosis, osteoarthritis / degenerative arthritis) | No / Yes | id. |
| 26 | Chronic joint inflammation (arthritis) | No / Yes | id. |
| 27 | Loss of bone tissue (osteoporosis) | No / Yes | id. |
| 28 | Hip fracture or other bone fractures | No / Yes | id. |
| 29 | Dizziness | No / Yes | id. |
| 30 | Neurological disease (Parkinson’s disease, multiple sclerosis, epilepsy) | No / Yes | id. |
| 31 | Depression | No / Yes | id. |
| 32 | Anxiety / panic disorder | No / Yes | id. |
| 33 | Dementia | No / Yes | id. |
| 34 | Hearing problems (despite hearing aid) | No / Yes | id. |
| 35 | Problems with vision (despite glasses / contact lenses) | No / Yes | id. |
| 36 | Did somebody help you with this questionnaire? | No / Yes | id. |
| 37 | If so, what type of help did you get?. | I selected the answers, and someone else wrote them down / I selected the answers together with someone else, who wrote them down / Someone else selected the answers for me and wrote them down | Not included in FI |

The Frailty Index was calculated as the sum of the values for each question, divided by the number of questions answered. The ADL score was determined by counting the number of Questions 4 through 9 with answers not equivalent to ‘Yes, without any effort’. The IADL score was determined by counting the number of Questions 10 through 13 with answers not equivalent to ‘Yes, without any effort’.
