# Supplementary material for: Quality of Care Perceived by Older Patients and Caregivers in Integrated Care Pathways With Interviewing Assistance From a Social Robot: Noninferiority Randomized Controlled Trial
Source: J Med Internet Res. 2020 Sep 9;22(9):e18787. doi: 10.2196/18787 (PMC7511864; doi:10.2196/18787)
Supplement: Multimedia Appendix 3 [file jmir_v22i9e18787_app3.docx]

# Multimedia Appendix 3 – Detailed description of the robot-patient interaction

The healthcare professional invited the patient and caregiver to sit in front of the robot. The healthcare professional then initiated a short dialogue with the robot, including the entering of the patient ID number and some other formal data. This took approximately one minute. The healthcare professional also informed the patient that the robot could not state a question and listen at the same time, so patients were asked to delay answering until it completed pronouncing the question and a blue bar appeared on the screen. The patient could then start the interview by saying ‘start’ to the robot, which then explained the interview procedure. It then asked whether the explanation should be repeated. If not, the robot started the questioning. Each time the robot finished a question, the patient had four answer options: to give one of the possible default answers, to ask for an explanation of the question, to skip a question or to change the answer of the previous question. At the end of the interview, the robot displayed a questionnaire report on its screen with answers that required the attention of the healthcare professional, as well as the calculated Frailty Index, Activities of Daily Living (ADL) score and Instrumental Activities of Daily Living (IADL) score (see Multimedia Appendix 1). This information served as input for the subsequent interaction between the patient and the healthcare professional for purposes of shared decision-making (1).

For each question, the default answer set was divided into two groups: 1) answers indicating serious conditions, which could possibly invoke empathy on the part of a healthcare professional; and 2) answers indicating minor conditions, which would not require separate discussion. If a burdensome condition was disclosed, the robot could give an empathic statement. It did not always do so, however, as pilot testing had indicated that excessively frequent empathic statements were perceived as annoying rather than comforting. An example of such an empathic statement was “That must be difficult for you” or “I am sorry to hear that”. This empathic statement was followed by a ‘Trust’ statement, which was implemented by statements referring to the healthcare professional, who would return to robot-selected issues later. For example: “Dr Jones will discuss this with you” or “Dr Jones will get back to you on this.” When the patient gave an answer indicating a minor condition, the robot only repeated the answer and continued with the next question.

Human-interface design standards for older adults were used for the display layout (minimalistic design, i.e. large fonts on an empty background) (2). Based on previous experience, robot voice speed was set to 80% of the default speed (3). The robot was further programmed to make specially designed body motions and arm gestures that are consistent with a professional questioning dialogue.


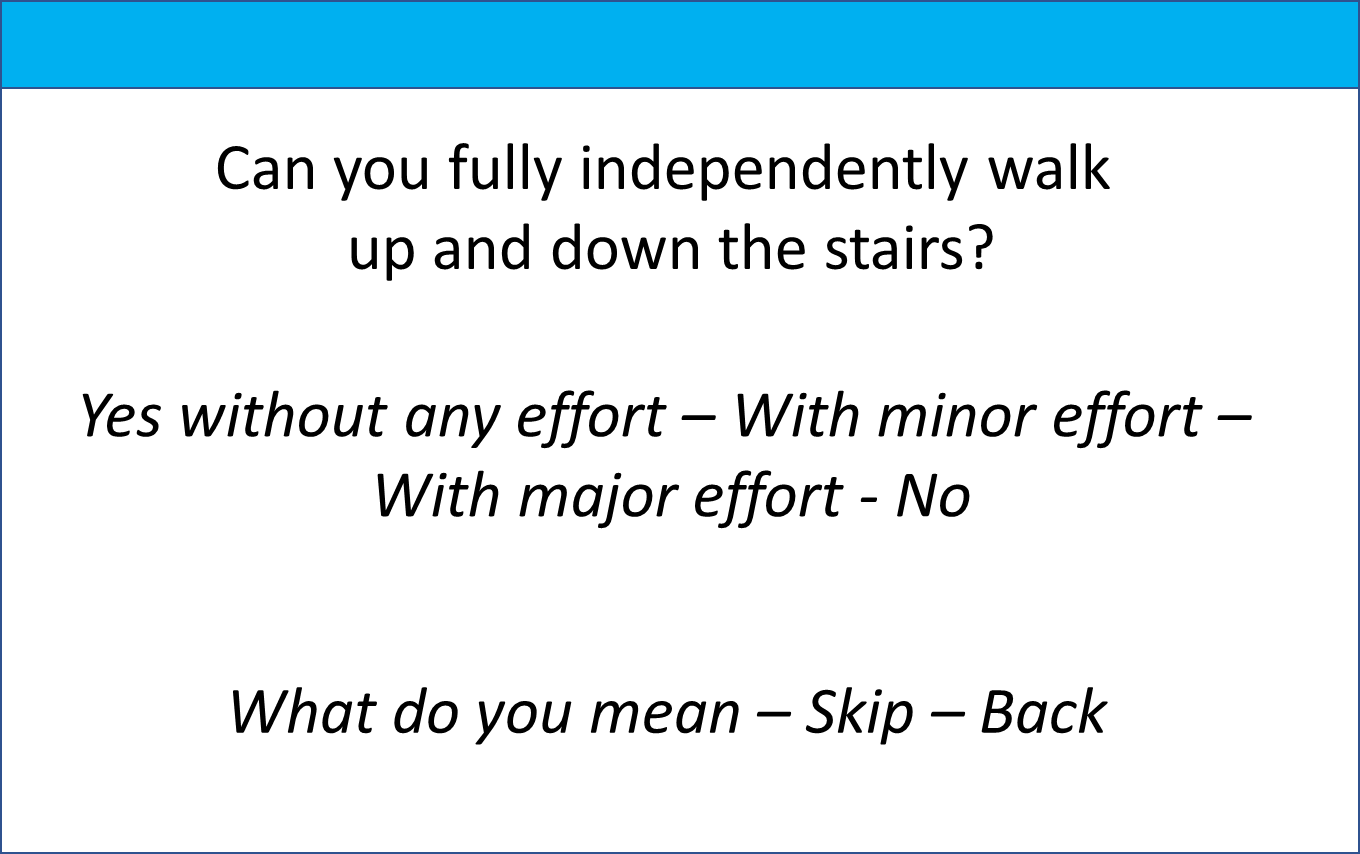


Figure MA3-1 - Screen layout

# References

1. Jamieson T, Goldfarb A. Clinical considerations when applying machine learning to decision-support tasks versus automation. 2019;

2. Czaja SJ, Boot WR, Charness N, Rogers WA. Designing for Older Adults - Principles and Creative Human Factors Approaches, Third Edition. Taylor & Francis Ltd; 2019. 276 p.

3. Boumans R, van Meulen F, Hindriks K, Neerincx M, Olde Rikkert M. A Feasibility Study of a Social Robot Collecting Patient Reported Outcome Measurements from Older Adults. Int J of Soc Robotics 12, 259-266 (2020). [doi: 10.1007/s12369-019-00561-8]
