# Supplementary material for: Quality of Care Perceived by Older Patients and Caregivers in Integrated Care Pathways With Interviewing Assistance From a Social Robot: Noninferiority Randomized Controlled Trial
Source: J Med Internet Res. 2020 Sep 9;22(9):e18787. doi: 10.2196/18787 (PMC7511864; doi:10.2196/18787)
Supplement: Multimedia Appendix 5 [file jmir_v22i9e18787_app5.docx]

# Multimedia Appendix 5 – CQI questions

Table MA5-1 - CQI questions

| Questions |
| --- |
| *Introductory questions.* |
| 1. Did you feel welcome at the outpatient clinic? |
| 2. Was the person who received you helpful? |
| 3. Did the outpatient clinic offer enough privacy? |
| *You have been in contact with a care provider (doctor, physician assistant, nurse or robot) who has supervised you.*  4. Was this care provider helpful? |
| 5. Did this care provider take you seriously? |
| 6. Did this care provider listen to you carefully? |
| 7. Did this care provider have enough time for you? |
| 8. Was this care provider competent?  *The following questions are about your visit to the outpatient clinic in general.* |
| 9. What rating would you give the outpatient clinic? |
| 10. Would you recommend this outpatient clinic to your family and friends? |
