# Supplementary material for: Quality of Care Perceived by Older Patients and Caregivers in Integrated Care Pathways With Interviewing Assistance From a Social Robot: Noninferiority Randomized Controlled Trial
Source: J Med Internet Res. 2020 Sep 9;22(9):e18787. doi: 10.2196/18787 (PMC7511864; doi:10.2196/18787)
Supplement: Multimedia Appendix 6 [file jmir_v22i9e18787_app6.docx]

# Multimedia Appendix 6 – Observation form

| Question | Answer scale | Exchange of information between patient and caregiver | Other event happening during the robot-patient interaction |
| --- | --- | --- | --- |
| 1. On a scale from 0 to 10, how do you rate your health? | 0–10 | - / + / ++ |  |

Figure MA6-1 – Observation form row (repeated for all questions).
