# Supplementary material for: Quality of Care Perceived by Older Patients and Caregivers in Integrated Care Pathways With Interviewing Assistance From a Social Robot: Noninferiority Randomized Controlled Trial
Source: J Med Internet Res. 2020 Sep 9;22(9):e18787. doi: 10.2196/18787 (PMC7511864; doi:10.2196/18787)
Supplement: Multimedia Appendix 7 [file jmir_v22i9e18787_app7.docx]

# Multimedia Appendix 7 – Robot-usability questions

Table MA7-1 – Questions on patient and caregiver’s opinion on robot usability

| Question | Variable |
| --- | --- |
| Did you like answering the robot’s questions? | Perceived Enjoyment |
| Did you have sufficient time to answer the questions? | Perceived Ease of Use |
| Did you find it easy to answer the robot’s questions? | Perceived Ease of Use |
| Did the robot respond correctly to your answers? | Trust |
