# Supplementary material for: Quality of Care Perceived by Older Patients and Caregivers in Integrated Care Pathways With Interviewing Assistance From a Social Robot: Noninferiority Randomized Controlled Trial
Source: J Med Internet Res. 2020 Sep 9;22(9):e18787. doi: 10.2196/18787 (PMC7511864; doi:10.2196/18787)
Supplement: Multimedia Appendix 8 [file jmir_v22i9e18787_app8.docx]

# Multimedia Appendix 8 – Baseline tables

The tables in this appendix show the baseline demographic and clinical characteristics for each group.

Table MA8-1 - Demographics and main clinical parameters of the intervention and control group

|  | With robot |  | Control group |  |
| --- | --- | --- | --- | --- |
|  | Mean | SD | Mean | SD |
| Age (yrs) | 78.14 | 6.96 | 76.66 | 7.66 |
| General health score* | 7.14 | 1.46 | 7.00 | 1.89 |
| Frailty index** | 0.25 | 0.15 | 0.26 | 0.15 |
| Quality of life* | 7.51 | 1.91 | 7.06 | 1.61 |

*range 0–10 **range 0–1

Table MA8-2 - Clinical characteristics of all participants (in frequencies)

|  |  | With robot | Control group |
| --- | --- | --- | --- |
| Male |  | 21 | 18 |
| Female |  | 16 | 20 |
| Memory complaints |  | 19 | 26 |
| Pain: | None | 11 | 14 |
|  | A little | 12 | 9 |
|  | Moderate | 8 | 10 |
|  | Severe | 6 | 3 |
|  | Extreme | 0 | 1 |
| Frailty value: | Robust | 3 | 1 |
|  | Pre-frail | 13 | 17 |
|  | Frail | 21 | 15 |
| Diabetes |  | 2 | 4 |
| Stroke |  | 8 | 7 |
| Heart failure |  | 13 | 14 |
| Cancer |  | 11 | 10 |
| Asthma |  | 11 | 5 |
| Incontinence |  | 7 | 8 |
| Arthrosis |  | 19 | 9 |
| Arthritis |  | 10 | 1 |
| Osteoporosis |  | 12 | 4 |
| Fractures |  | 3 | 8 |
| Dizziness |  | 9 | 11 |
| Neurological disease |  | 0 | 0 |
| Depression |  | 3 | 8 |
| Anxiety |  | 4 | 9 |
| Dementia |  | 11 | 10 |
| Hearing problems |  | 9 | 8 |
| Vision problems |  | 10 | 3 |
| Falling |  | 9 | 10 |
