# Supplementary material for: Quality of Care Perceived by Older Patients and Caregivers in Integrated Care Pathways With Interviewing Assistance From a Social Robot: Noninferiority Randomized Controlled Trial
Source: J Med Internet Res. 2020 Sep 9;22(9):e18787. doi: 10.2196/18787 (PMC7511864; doi:10.2196/18787)
Supplement: Multimedia Appendix 9 [file jmir_v22i9e18787_app9.docx]

# Multimedia Appendix 9 – Consumer Quality Indexes, by question

Table MA9-1 - Consumer Quality Indexes - Scores by question (range 0-10)

|  | Patient | | | | Informal caregiver | | | |
| --- | --- | --- | --- | --- | --- | --- | --- | --- |
|  | With robot | | Control group | | With robot | | Control group | |
|  | Mean | SD | Mean | SD | Mean | SD | Mean | SD |
| 1. Did you feel welcome at the outpatient clinic? | 9.24 | 0.90 | 8.84 | 1.10 | 9.00 | 1.05 | 9.09 | 0.79 |
| 2. Was the person who received you helpful? | 9.05 | 1.22 | 8.89 | 0.98 | 9.17 | 0.91 | 9.20 | 0.72 |
| 3. Did the outpatient clinic offer sufficient privacy? | 9.28 | 0.82 | 8.86 | 1.08 | 9.24 | 0.79 | 8.91 | 0.74 |
| You have been in contact with a care provider (doctor, physician assistant, nurse) who has supervised you. |  |  |  |  |  |  |  |  |
| 4. Was this care provider helpful? | 9.24 | 0.90 | 8.89 | 0.89 | 9.27 | 0.91 | 9.14 | 0.63 |
| 5. Did this care provider take you seriously? | 9.35 | 0.79 | 9.16 | 0.75 | 9.30 | 0.88 | 9.29 | 0.71 |
| 6. Did this care provider listen to you carefully? | 9.46 | 0.69 | 9.11 | 0.76 | 9.30 | 0.88 | 9.20 | 0.76 |
| 7. Did this care provider have enough time for you? | 9.54 | 0.56 | 9.13 | 0.70 | 9.40 | 0.72 | 9.09 | 0.74 |
| 8. Was this care provider competent? | 9.51 | 0.61 | 9.24 | 0.79 | 9.34 | 0.90 | 9.21 | 0.70 |
| The following questions are about your visit to the outpatient clinic in general. |  |  |  |  |  |  |  |  |
| 9. What rating would you give the outpatient clinic? | 8.86 | 1.03 | 8.84 | 0.87 | 9.03 | 0.82 | 8.82 | 0.80 |
| 10. Would you recommend this outpatient clinic to your family and friends? | 9.19 | 0.85 | 8.97 | 0.82 | 9.10 | 0.82 | 9.03 | 0.82 |
