# Supplementary material for: Quality of Care Perceived by Older Patients and Caregivers in Integrated Care Pathways With Interviewing Assistance From a Social Robot: Noninferiority Randomized Controlled Trial
Source: J Med Internet Res. 2020 Sep 9;22(9):e18787. doi: 10.2196/18787 (PMC7511864; doi:10.2196/18787)
Supplement: Multimedia Appendix 10 [file jmir_v22i9e18787_app10.docx]

# Multimedia Appendix 10 – CQI distributions


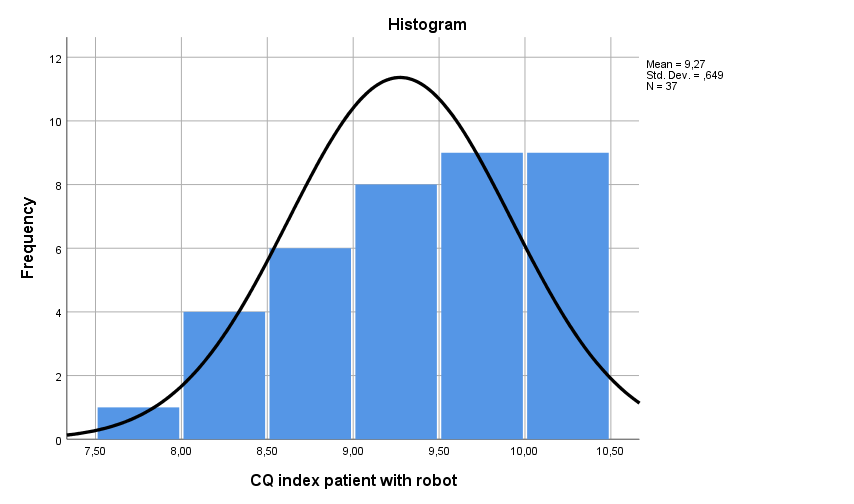


Figure MA10-1 - CQI distribution in the intervention group (N=37)


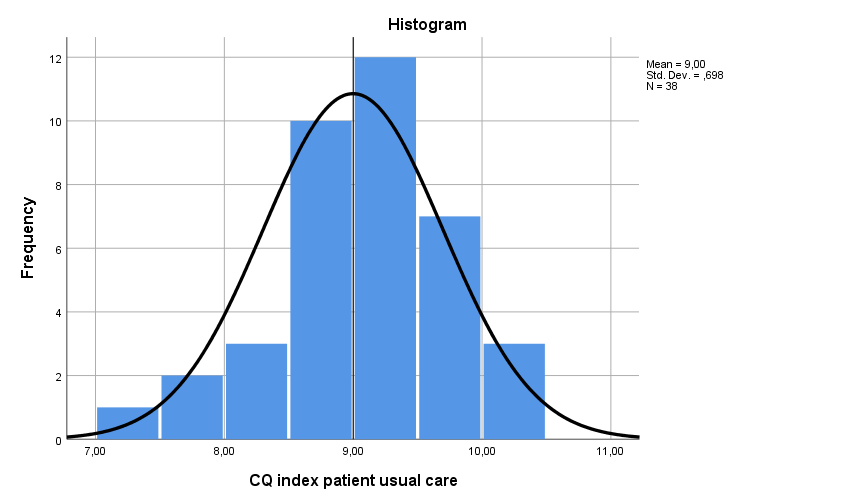


Figure MA10-2 - CQI distribution in the control group (N=38)
