# Supplementary material for: Quality of Care Perceived by Older Patients and Caregivers in Integrated Care Pathways With Interviewing Assistance From a Social Robot: Noninferiority Randomized Controlled Trial
Source: J Med Internet Res. 2020 Sep 9;22(9):e18787. doi: 10.2196/18787 (PMC7511864; doi:10.2196/18787)
Supplement: Multimedia Appendix 11 [file jmir_v22i9e18787_app11.docx]

# Multimedia Appendix 11 - Patient and caregiver opinion on robot usability

Table MA11-1 - Patient and caregiver opinion on robot usability on a scale from 0 (very low) to 10 (excellent)

|  |  | Patient (n=37) | | Caregiver (n=24) | |
| --- | --- | --- | --- | --- | --- |
|  | Variable | Mean | SD | Mean | SD |
| Did you like answering the robot’s questions? | Perceived Enjoyment | 7.81 | 2.01 | 7.55 | 2.11 |
| Did you have sufficient time to answer the questions? | Perceived Ease of Use | 8.51 | 1.63 | 8.45 | 1.10 |
| Did you find it easy to answer the robot’s questions? | Perceived Ease of Use | 8.11 | 1.89 | 7.86 | 1.67 |
| Did the robot respond correctly to your answers? | Trust | 8.43 | 1.38 | 7.59 | 1.76 |
