# Supplementary material for: Quality of Care Perceived by Older Patients and Caregivers in Integrated Care Pathways With Interviewing Assistance From a Social Robot: Noninferiority Randomized Controlled Trial
Source: J Med Internet Res. 2020 Sep 9;22(9):e18787. doi: 10.2196/18787 (PMC7511864; doi:10.2196/18787)
Supplement: Multimedia Appendix 12 [file jmir_v22i9e18787_app12.pdf]

CONSORT-EHEALTH (V 1.6.1) - Submission/Publication Form

The CONSORT-EHEALTH checklist is intended for authors of randomized trials evaluating web-based and Internet-based applications/interventions, including mobile interventions, electronic games (incl multiplayer games), social media, certain telehealth applications, and other interactive and/or networked electronic applications. Some of the items (e.g. all subitems under item 5 - description of the intervention) may also be applicable for other study designs.

The goal of the CONSORT EHEALTH checklist and guideline is to be
a) a guide for reporting for authors of RCTs,
b) to form a basis for appraisal of an ehealth trial (in terms of validity)

CONSORT-EHEALTH items/subitems are MANDATORY reporting items for studies published in the Journal of Medical Internet Research and other journals / scientific societies endorsing the checklist.

Items numbered 1., 2., 3., 4a., 4b etc are original CONSORT or CONSORT-NPT (non-pharmacologic treatment) items.
Items with Roman numerals (i., ii, iii, iv etc.) are CONSORT-EHEALTH extensions/clarifications.

As the CONSORT-EHEALTH checklist is still considered in a formative stage, we would ask that you also RATE ON A SCALE OF 1-5 how important/useful you feel each item is FOR THE PURPOSE OF THE CHECKLIST and reporting guideline (optional).

Mandatory reporting items are marked with a red *.
In the textboxes, either copy & paste the relevant sections from your manuscript into this form - please include any quotes from your manuscript in QUOTATION MARKS,
or answer directly by providing additional information not in the manuscript, or elaborating on why the item was not relevant for this study.

YOUR ANSWERS WILL BE PUBLISHED AS A SUPPLEMENTARY FILE TO YOUR PUBLICATION IN JMIR AND ARE CONSIDERED PART OF YOUR PUBLICATION (IF ACCEPTED).
Please fill in these questions diligently. Information will not be copyedited, so please use proper spelling and grammar, use correct capitalization, and avoid abbreviations.

DO NOT FORGET TO SAVE AS PDF _AND_ CLICK THE SUBMIT BUTTON SO YOUR ANSWERS ARE IN OUR DATABASE !!!

Citation Suggestion (if you append the pdf as Appendix we suggest to cite this paper in the caption):
Eysenbach G, CONSORT-EHEALTH Group
CONSORT-EHEALTH: Improving and Standardizing Evaluation Reports of Web-based and Mobile Health Interventions
J Med Internet Res 2011;13(4):e126
URL: http://www.jmir.org/2011/4/e126/

doi: 10.2196/jmir.1923
PMID: 22209829

* Required

Your name *

First Last

Roel Boumans

Primary Affiliation (short), City, Country *

University of Toronto, Toronto, Canada

Radboud University Medical Center

Your e-mail address *

abc@gmail.com

roel.boumans@radboudumc.nl

Title of your manuscript *

Provide the (draft) title of your manuscript.

Quality of Care Perceived by Older Patients and Caregivers Does Not Suffer from Social Robot Assistance: A Noninferiority Randomised Controlled Trial

Name of your App/Software/Intervention *

If there is a short and a long/alternate name, write the short name first and add the long name in brackets.

Social Robot Pepper

Evaluated Version (if any)

e.g. "V1", "Release 2017-03-01", "Version 2.0.27913"

1.8a

Language(s) *

What language is the intervention/app in? If multiple languages are available, separate by comma (e.g. "English, French")

Dutch

URL of your Intervention Website or App

e.g. a direct link to the mobile app on app in appstore (itunes, Google Play), or URL of the website. If the intervention is a DVD or hardware, you can also link to an Amazon page.

Your answer

URL of an image/screenshot (optional)

Your answer

Accessibility *

Can an enduser access the intervention presently?

- ○ access is free and open
- ○ access only for special usergroups, not open
- ○ access is open to everyone, but requires payment/subscription/in-app purchases
- ○ app/intervention no longer accessible
- ◉ Other: The intervention is implemented in a robot

Primary Medical Indication/Disease/Condition *

e.g. "Stress", "Diabetes", or define the target group in brackets after the condition, e.g. "Autism (Parents of children with)", "Alzheimers (Informal Caregivers of)"

Frail older adults

Primary Outcomes measured in trial *

comma-separated list of primary outcomes reported in the trial

Consumer Quality Index questions

Secondary/other outcomes

Are there any other outcomes the intervention is expected to affect?

Time duration of the intervention

Recommended "Dose" *

What do the instructions for users say on how often the app should be used?

- ○ Approximately Daily
- ○ Approximately Weekly
- ○ Approximately Monthly
- ○ Approximately Yearly
- ◉ "as needed"
- ○ Other:

Approx. Percentage of Users (starters) still using the app as recommended after 3 months *

- (selected) unknown / not evaluated
- 0-10%
- 11-20%
- 21-30%
- 31-40%
- 41-50%
- 51-60%
- 61-70%
- 71%-80%
- 81-90%
- 91-100%
- Other:

Overall, was the app/intervention effective? *

- yes: all primary outcomes were significantly better in intervention group vs control
- partly: SOME primary outcomes were significantly better in intervention group vs control
- (selected) no statistically significant difference between control and intervention
- potentially harmful: control was significantly better than intervention in one or more outcomes
- inconclusive: more research is needed
- Other:

Article Preparation Status/Stage *

At which stage in your article preparation are you currently (at the time you fill in this form)

- ○ not submitted yet - in early draft status
- ○ not submitted yet - in late draft status, just before submission
- ◉ submitted to a journal but not reviewed yet
- ○ submitted to a journal and after receiving initial reviewer comments
- ○ submitted to a journal and accepted, but not published yet
- ○ published
- ○ Other:

Journal *

If you already know where you will submit this paper (or if it is already submitted), please provide the journal name (if it is not JMIR, provide the journal name under "other")

- ○ not submitted yet / unclear where I will submit this
- ◉ Journal of Medical Internet Research (JMIR)
- ○ JMIR mHealth and UHealth
- ○ JMIR Serious Games
- ○ JMIR Mental Health
- ○ JMIR Public Health
- ○ JMIR Formative Research
- ○ Other JMIR sister journal
- ○ Other:

Is this a full powered effectiveness trial or a pilot/feasibility trial? *

- ◯ Pilot/feasibility
- ◉ Fully powered

Manuscript tracking number *

If this is a JMIR submission, please provide the manuscript tracking number under "other" (The ms tracking number can be found in the submission acknowledgement email, or when you login as author in JMIR. If the paper is already published in JMIR, then the ms tracking number is the four-digit number at the end of the DOI, to be found at the bottom of each published article in JMIR)

- ◯ no ms number (yet) / not (yet) submitted to / published in JMIR
- ◯ Other:

TITLE AND ABSTRACT

1a) TITLE: Identification as a randomized trial in the title

1a) Does your paper address CONSORT item 1a? *

I.e does the title contain the phrase "Randomized Controlled Trial"? (if not, explain the reason under "other")

- ◉ yes
- ◯ Other:

1a-i) Identify the mode of delivery in the title

Identify the mode of delivery. Preferably use “web-based” and/or “mobile” and/or “electronic game” in the title. Avoid ambiguous terms like “online”, “virtual”, “interactive”. Use “Internet-based” only if Intervention includes non-web-based Internet components (e.g. email), use “computer-based” or “electronic” only if offline products are used. Use “virtual” only in the context of “virtual reality” (3-D worlds). Use “online” only in the context of “online support groups”. Complement or substitute product names with broader terms for the class of products (such as “mobile” or “smart phone” instead of “iphone”), especially if the application runs on different platforms.

	1	2	3	4	5	
subitem not at all important	○	○	○	○	◉	essential

Does your paper address subitem 1a-i? *

Copy and paste relevant sections from manuscript title (include quotes in quotation marks "like this" to indicate direct quotes from your manuscript), or elaborate on this item by providing additional information not in the ms, or briefly explain why the item is not applicable/relevant for your study

Social Robot

1a-ii) Non-web-based components or important co-interventions in title

Mention non-web-based components or important co-interventions in title, if any (e.g., “with telephone support”).

	1	2	3	4	5	
subitem not at all important	◉	○	○	○	○	essential

Does your paper address subitem 1a-ii?

Copy and paste relevant sections from manuscript title (include quotes in quotation marks "like this" to indicate direct quotes from your manuscript), or elaborate on this item by providing additional information not in the ms, or briefly explain why the item is not applicable/relevant for your study

Not applicable, intervention by social robot

1a-iii) Primary condition or target group in the title

Mention primary condition or target group in the title, if any (e.g., “for children with Type I Diabetes”)
Example: A Web-based and Mobile Intervention with Telephone Support for Children with Type I Diabetes: Randomized Controlled Trial

	1	2	3	4	5	
subitem not at all important	○	○	○	○	◉	essential

Does your paper address subitem 1a-iii? *

Copy and paste relevant sections from manuscript title (include quotes in quotation marks "like this" to indicate direct quotes from your manuscript), or elaborate on this item by providing additional information not in the ms, or briefly explain why the item is not applicable/relevant for your study

Older Patients and Caregivers

1b) ABSTRACT: Structured summary of trial design, methods, results, and conclusions

NPT extension: Description of experimental treatment, comparator, care providers, centers, and blinding status.

1b-i) Key features/functionalities/components of the intervention and comparator in the METHODS section of the ABSTRACT

Mention key features/functionalities/components of the intervention and comparator in the abstract. If possible, also mention theories and principles used for designing the site. Keep in mind the needs of systematic reviewers and indexers by including important synonyms. (Note: Only report in the abstract what the main paper is reporting. If this information is missing from the main body of text, consider adding it)

	1	2	3	4	5	
subitem not at all important	○	○	○	○	◉	essential

Does your paper address subitem 1b-i? *

Copy and paste relevant sections from the manuscript abstract (include quotes in quotation marks "like this" to indicate direct quotes from your manuscript), or elaborate on this item by providing additional information not in the ms, or briefly explain why the item is not applicable/relevant for your study

Multicentre two parallel groups, non-blinded, randomised controlled trial testing for non-inferiority of the quality of care delivered through robot-assisted care.

1b-ii) Level of human involvement in the METHODS section of the ABSTRACT

Clarify the level of human involvement in the abstract, e.g., use phrases like “fully automated” vs. “therapist/nurse/care provider/physician-assisted” (mention number and expertise of providers involved, if any). (Note: Only report in the abstract what the main paper is reporting. If this information is missing from the main body of text, consider adding it)

	1	2	3	4	5	
subitem not at all important	○	○	○	○	●	essential

Does your paper address subitem 1b-ii?

Copy and paste relevant sections from the manuscript abstract (include quotes in quotation marks "like this" to indicate direct quotes from your manuscript), or elaborate on this item by providing additional information not in the ms, or briefly explain why the item is not applicable/relevant for your study

The setting concerned two outpatient clinics in the period July–December 2019. Of 419 subsequent patients visiting the participating outpatient clinics, 110 older patients fit the recruitment criteria

1b-iii) Open vs. closed, web-based (self-assessment) vs. face-to-face assessments in the METHODS section of the ABSTRACT

Mention how participants were recruited (online vs. offline), e.g., from an open access website or from a clinic or a closed online user group (closed usergroup trial), and clarify if this was a purely web-based trial, or there were face-to-face components (as part of the intervention or for assessment). Clearly say if outcomes were self-assessed through questionnaires (as common in web-based trials). Note: In traditional offline trials, an open trial (open-label trial) is a type of clinical trial in which both the researchers and participants know which treatment is being administered. To avoid confusion, use "blinded" or "unblinded" to indicated the level of blinding instead of "open", as "open" in web-based trials usually refers to "open access" (i.e. participants can self-enrol). (Note: Only report in the abstract what the main paper is reporting. If this information is missing from the main body of text, consider adding it)

	1	2	3	4	5	
subitem not at all important	○	○	○	○	◉	essential

Does your paper address subitem 1b-iii?

Copy and paste relevant sections from the manuscript abstract (include quotes in quotation marks "like this" to indicate direct quotes from your manuscript), or elaborate on this item by providing additional information not in the ms, or briefly explain why the item is not applicable/relevant for your study

From manuscript: Patients were recruited from the group of patients scheduled to visit the outpatient clinics of the Geriatrics departments of both hospitals.

1b-iv) RESULTS section in abstract must contain use data

Report number of participants enrolled/assessed in each group, the use/uptake of the intervention (e.g., attrition/adherence metrics, use over time, number of logins etc.), in addition to primary/secondary outcomes. (Note: Only report in the abstract what the main paper is reporting. If this information is missing from the main body of text, consider adding it)

	1	2	3	4	5	
subitem not at all important	○	○	○	○	◉	essential

Does your paper address subitem 1b-iv?

Copy and paste relevant sections from the manuscript abstract (include quotes in quotation marks "like this" to indicate direct quotes from your manuscript), or elaborate on this item by providing additional information not in the ms, or briefly explain why the item is not applicable/relevant for your study

110 older patients fit the recruitment criteria

1b-v) CONCLUSIONS/DISCUSSION in abstract for negative trials

Conclusions/Discussions in abstract for negative trials: Discuss the primary outcome - if the trial is negative (primary outcome not changed), and the intervention was not used, discuss whether negative results are attributable to lack of uptake and discuss reasons. (Note: Only report in the abstract what the main paper is reporting. If this information is missing from the main body of text, consider adding it)

	1	2	3	4	5	
subitem not at all important	○	○	○	○	●	essential

Does your paper address subitem 1b-v?

Copy and paste relevant sections from the manuscript abstract (include quotes in quotation marks "like this" to indicate direct quotes from your manuscript), or elaborate on this item by providing additional information not in the ms, or briefly explain why the item is not applicable/relevant for your study

There was no significant difference in the total patient CQI scores of the patients with the robot pathway (M=9.19, SD=0.83, n=38) and those in the control group (M=9.00, SD=0.70, n=38); P=.29, 95% CI (-0.16 to 0.54), and no significant difference for their caregivers (intervention group M=9.15, SD=0.78, n=32; control group M=9.12, SD=0.61, n=36); P=.85, 95% CI (-0.31 to 0.37).

INTRODUCTION

2a) In INTRODUCTION: Scientific background and explanation of rationale

2a-i) Problem and the type of system/solution

Describe the problem and the type of system/solution that is object of the study: intended as stand-alone intervention vs. incorporated in broader health care program? Intended for a particular patient population? Goals of the intervention, e.g., being more cost-effective to other interventions, replace or complement other solutions? (Note: Details about the intervention are provided in “Methods” under 5)

	1	2	3	4	5	
subitem not at all important	○	○	○	○	●	essential

Does your paper address subitem 2a-i? *

Copy and paste relevant sections from the manuscript (include quotes in quotation marks "like this" to indicate direct quotes from your manuscript), or elaborate on this item by providing additional information not in the ms, or briefly explain why the item is not applicable/relevant for your study

The intervention was incorporated in the outpatient clinic's Integrated Care Pathway for the treatment and monitoring of patients with cognitive decline

2a-ii) Scientific background, rationale: What is known about the (type of) system

Scientific background, rationale: What is known about the (type of) system that is the object of the study (be sure to discuss the use of similar systems for other conditions/diagnoses, if appropiate), motivation for the study, i.e. what are the reasons for and what is the context for this specific study, from which stakeholder viewpoint is the study performed, potential impact of findings [2]. Briefly justify the choice of the comparator.

	1	2	3	4	5	
subitem not at all important	○	○	○	◉	○	essential

Does your paper address subitem 2a-ii? *

Copy and paste relevant sections from the manuscript (include quotes in quotation marks "like this" to indicate direct quotes from your manuscript), or elaborate on this item by providing additional information not in the ms, or briefly explain why the item is not applicable/relevant for your study

Many studies have been conducted on assistive robots for healthcare professionals, as well as on the cost-effectiveness of care pathways. For example, DiNuovo et al. used the social robot Pepper to study the assessment of cognitive skills with the Montreal Cognitive Assessment (MoCA) of university personnel[8–10]. Bandera et al. designed CLARC, a robot designed to perform a Comprehensive Geriatric Assessment, but have not yet published results on its interviewing performance[11–13]. Broadbent et al. used a robot to provide at-home assistance to people with COPD. This robot spoke, but could not listen, and patients entered their responses on a touch screen[14]. D'Onofrio et al. describe the MARIO robot designed for the practical daily living support of people with dementia in nursing homes, focusing on differences in feasibility between UK, Ireland and Sweden[15].

2b) In INTRODUCTION: Specific objectives or hypotheses

Does your paper address CONSORT subitem 2b? *

Copy and paste relevant sections from the manuscript (include quotes in quotation marks "like this" to indicate direct quotes from your manuscript), or elaborate on this item by providing additional information not in the ms, or briefly explain why the item is not applicable/relevant for your study

Our hypothesis is that the quality of care perceived by patients and caregivers in a pathway that includes a social robot for a standardised part of nurse-patient dialogue is not significantly lower than that perceived by the control group, whose pathway involved the continued presence of healthcare professionals (a non-inferiority hypothesis).

METHODS

3a) Description of trial design (such as parallel, factorial) including allocation ratio

Does your paper address CONSORT subitem 3a? *

Copy and paste relevant sections from the manuscript (include quotes in quotation marks "like this" to indicate direct quotes from your manuscript), or elaborate on this item by providing additional information not in the ms, or briefly explain why the item is not applicable/relevant for your study

The study was designed as a between-subject, multi-center randomised controlled trial among patients visiting the outpatient memory clinics at the two teaching hospitals XXX and YYY.

3b) Important changes to methods after trial commencement (such as eligibility criteria), with reasons

Does your paper address CONSORT subitem 3b? *

Copy and paste relevant sections from the manuscript (include quotes in quotation marks "like this" to indicate direct quotes from your manuscript), or elaborate on this item by providing additional information not in the ms, or briefly explain why the item is not applicable/relevant for your study

Not applicable, no changes

3b-i) Bug fixes, Downtimes, Content Changes

Bug fixes, Downtimes, Content Changes: ehealth systems are often dynamic systems. A description of changes to methods therefore also includes important changes made on the intervention or comparator during the trial (e.g., major bug fixes or changes in the functionality or content) (5-iii) and other “unexpected events” that may have influenced study design such as staff changes, system failures/downtimes, etc. [2].

	1	2	3	4	5	
subitem not at all important	○	○	○	○	○	essential

Does your paper address subitem 3b-i?

Copy and paste relevant sections from the manuscript (include quotes in quotation marks "like this" to indicate direct quotes from your manuscript), or elaborate on this item by providing additional information not in the ms, or briefly explain why the item is not applicable/relevant for your study

Not applicable, no major bug fixes necessary

4a) Eligibility criteria for participants

Does your paper address CONSORT subitem 4a? *

Copy and paste relevant sections from the manuscript (include quotes in quotation marks "like this" to indicate direct quotes from your manuscript), or elaborate on this item by providing additional information not in the ms, or briefly explain why the item is not applicable/relevant for your study

Inclusion criteria were the ability to speak and read Dutch, and being assisted by one of the regular staff nurses or physicians taking part in the study. Exclusion criteria were serious hearing or vision problems, serious cognitive problems, paranoia or similar psychiatric problems, as well as situations in which the patient had previously been asked to complete the Topic-SF questionnair.

4a-i) Computer / Internet literacy

Computer / Internet literacy is often an implicit “de facto” eligibility criterion - this should be explicitly clarified.

	1	2	3	4	5	
subitem not at all important	◉	○	○	○	○	essential

Does your paper address subitem 4a-i?

Copy and paste relevant sections from the manuscript (include quotes in quotation marks "like this" to indicate direct quotes from your manuscript), or elaborate on this item by providing additional information not in the ms, or briefly explain why the item is not applicable/relevant for your study

Not applicable. Intervention (with social robot) did not require any computer or internet literacy.

4a-ii) Open vs. closed, web-based vs. face-to-face assessments:

Open vs. closed, web-based vs. face-to-face assessments: Mention how participants were recruited (online vs. offline), e.g., from an open access website or from a clinic, and clarify if this was a purely web-based trial, or there were face-to-face components (as part of the intervention or for assessment), i.e., to what degree got the study team to know the participant. In online-only trials, clarify if participants were quasi-anonymous and whether having multiple identities was possible or whether technical or logistical measures (e.g., cookies, email confirmation, phone calls) were used to detect/prevent these.

	1	2	3	4	5	
subitem not at all important	○	○	○	◉	○	essential

Does your paper address subitem 4a-ii? *

Copy and paste relevant sections from the manuscript (include quotes in quotation marks "like this" to indicate direct quotes from your manuscript), or elaborate on this item by providing additional information not in the ms, or briefly explain why the item is not applicable/relevant for your study

Patients were recruited from the group of patients scheduled to visit the outpatient clinics of the Geriatrics departments of both hospitals.

4a-iii) Information giving during recruitment

Information given during recruitment. Specify how participants were briefed for recruitment and in the informed consent procedures (e.g., publish the informed consent documentation as appendix, see also item X26), as this information may have an effect on user self-selection, user expectation and may also bias results.

	1	2	3	4	5	
subitem not at all important	○	○	○	◉	○	essential

Does your paper address subitem 4a-iii?

Copy and paste relevant sections from the manuscript (include quotes in quotation marks "like this" to indicate direct quotes from your manuscript), or elaborate on this item by providing additional information not in the ms, or briefly explain why the item is not applicable/relevant for your study

Patients were screened for exclusion criteria and consent was asked, all according to a standardised script. (This script included the provision of written en verbal information to the patient).

4b) Settings and locations where the data were collected

Does your paper address CONSORT subitem 4b? *

Copy and paste relevant sections from the manuscript (include quotes in quotation marks "like this" to indicate direct quotes from your manuscript), or elaborate on this item by providing additional information not in the ms, or briefly explain why the item is not applicable/relevant for your study

The setting concerned two outpatient clinics.

4b-i) Report if outcomes were (self-)assessed through online questionnaires

Clearly report if outcomes were (self-)assessed through online questionnaires (as common in web-based trials) or otherwise.

	1	2	3	4	5	
subitem not at all important	○	○	○	○	◉	essential

Does your paper address subitem 4b-i? *

Copy and paste relevant sections from the manuscript (include quotes in quotation marks "like this" to indicate direct quotes from your manuscript), or elaborate on this item by providing additional information not in the ms, or briefly explain why the item is not applicable/relevant for your study

As main outcome measure the Customised Consumer Quality Index (CQI) was used, as reported by patient and caregiver for the outpatient pathway of care.

4b-ii) Report how institutional affiliations are displayed

Report how institutional affiliations are displayed to potential participants [on ehealth media], as affiliations with prestigious hospitals or universities may affect volunteer rates, use, and reactions with regards to an intervention.(Not a required item – describe only if this may bias results)

	1	2	3	4	5	
subitem not at all important	●	○	○	○	○	essential

Does your paper address subitem 4b-ii?

Copy and paste relevant sections from the manuscript (include quotes in quotation marks "like this" to indicate direct quotes from your manuscript), or elaborate on this item by providing additional information not in the ms, or briefly explain why the item is not applicable/relevant for your study

Not applicable

5) The interventions for each group with sufficient details to allow replication, including how and when they were actually administered

5-i) Mention names, credential, affiliations of the developers, sponsors, and owners

Mention names, credential, affiliations of the developers, sponsors, and owners [6] (if authors/evaluators are owners or developer of the software, this needs to be declared in a “Conflict of interest” section or mentioned elsewhere in the manuscript).

	1	2	3	4	5	
subitem not at all important	○	○	○	●	○	essential

Does your paper address subitem 5-i?

Copy and paste relevant sections from the manuscript (include quotes in quotation marks "like this" to indicate direct quotes from your manuscript), or elaborate on this item by providing additional information not in the ms, or briefly explain why the item is not applicable/relevant for your study

The social robot used in this study was a Pepper robot, version 1.8a, using the Naoqi operating system, version 3.9, and manufactured by Softbank Robotics (Tokyo, Japan)[10]. The robot software necessary for the intervention was designed and programmed using Android Studio, version 3.1 (Google Inc, Mountain View, CA, USA) and Java, version 8 (Oracle Corp., Redwood Shores, CA, USA). (These institutions were not involved in the trial and the software was programmed by the researcher.)

5-ii) Describe the history/development process

Describe the history/development process of the application and previous formative evaluations (e.g., focus groups, usability testing), as these will have an impact on adoption/use rates and help with interpreting results.

	1	2	3	4	5	
subitem not at all important	○	○	◉	○	○	essential

Does your paper address subitem 5-ii?

Copy and paste relevant sections from the manuscript (include quotes in quotation marks "like this" to indicate direct quotes from your manuscript), or elaborate on this item by providing additional information not in the ms, or briefly explain why the item is not applicable/relevant for your study

Previous versions of the social robot intervention application have been evaluated among community dwelling older adults: "The opinions of the patients and caregivers concerning the robot were in line with previous findings on the positive appreciation results on robot interaction amongst community dwelling older adults[28,29], as well as with the results reported in our exploratory study amongst hospitalised patients[34]."

5-iii) Revisions and updating

Revisions and updating. Clearly mention the date and/or version number of the application/intervention (and comparator, if applicable) evaluated, or describe whether the intervention underwent major changes during the evaluation process, or whether the development and/or content was “frozen” during the trial. Describe dynamic components such as news feeds or changing content which may have an impact on the replicability of the intervention (for unexpected events see item 3b).

	1	2	3	4	5	
subitem not at all important	(●)	○	○	○	○	essential

Does your paper address subitem 5-iii?

Copy and paste relevant sections from the manuscript (include quotes in quotation marks "like this" to indicate direct quotes from your manuscript), or elaborate on this item by providing additional information not in the ms, or briefly explain why the item is not applicable/relevant for your study

The intervention has not undergone major changes during the evaluation process.

5-iv) Quality assurance methods

Provide information on quality assurance methods to ensure accuracy and quality of information provided [1], if applicable.

	1	2	3	4	5	
subitem not at all important	(●)	○	○	○	○	essential

Does your paper address subitem 5-iv?

Copy and paste relevant sections from the manuscript (include quotes in quotation marks "like this" to indicate direct quotes from your manuscript), or elaborate on this item by providing additional information not in the ms, or briefly explain why the item is not applicable/relevant for your study

Not applicable.

5-v) Ensure replicability by publishing the source code, and/or providing screenshots/screen-capture video, and/or providing flowcharts of the algorithms used

Ensure replicability by publishing the source code, and/or providing screenshots/screen-capture video, and/or providing flowcharts of the algorithms used. Replicability (i.e., other researchers should in principle be able to replicate the study) is a hallmark of scientific reporting.

	1	2	3	4	5	
subitem not at all important	○	○	○	○	◉	essential

Does your paper address subitem 5-v?

Copy and paste relevant sections from the manuscript (include quotes in quotation marks "like this" to indicate direct quotes from your manuscript), or elaborate on this item by providing additional information not in the ms, or briefly explain why the item is not applicable/relevant for your study

The interaction design has been described in the manuscript and in more detail in the appendices.

5-vi) Digital preservation

Digital preservation: Provide the URL of the application, but as the intervention is likely to change or disappear over the course of the years; also make sure the intervention is archived (Internet Archive, webcitation.org, and/or publishing the source code or screenshots/videos alongside the article). As pages behind login screens cannot be archived, consider creating demo pages which are accessible without login.

	1	2	3	4	5	
subitem not at all important	◉	○	○	○	○	essential

Does your paper address subitem 5-vi?

Copy and paste relevant sections from the manuscript (include quotes in quotation marks "like this" to indicate direct quotes from your manuscript), or elaborate on this item by providing additional information not in the ms, or briefly explain why the item is not applicable/relevant for your study

The application is implemented on a social robot.

5-vii) Access

Access: Describe how participants accessed the application, in what setting/context, if they had to pay (or were paid) or not, whether they had to be a member of specific group. If known, describe how participants obtained “access to the platform and Internet” [1]. To ensure access for editors/reviewers/readers, consider to provide a “backdoor” login account or demo mode for reviewers/readers to explore the application (also important for archiving purposes, see vi).

	1	2	3	4	5	
subitem not at all important	○	○	○	○	◉	essential

Does your paper address subitem 5-vii? *

Copy and paste relevant sections from the manuscript (include quotes in quotation marks "like this" to indicate direct quotes from your manuscript), or elaborate on this item by providing additional information not in the ms, or briefly explain why the item is not applicable/relevant for your study

The healthcare professional guided the patient from the waiting room to an examination room, where the robot was or was not present, depending on randomisation.
In the intervention pathway, the healthcare professional started the interview with several open-ended questions on the patient’s general health status. This was followed by the introduction of the robot, which subsequently conducted the TOPICS-SF questionnaire interview. Upon completing the interview, the robot generated a report of the PROM and Frailty Index results, including the (Instrumental) Activities of Daily Living scores. This report was input for subsequent interactions between the patient and the healthcare professional within the context of shared decision-making[21]. The robot-patient interaction is detailed in Appendix C.

5-viii) Mode of delivery, features/functionalities/components of the intervention and comparator, and the theoretical framework

Describe mode of delivery, features/functionalities/components of the intervention and comparator, and the theoretical framework [6] used to design them (instructional strategy [1], behaviour change techniques, persuasive features, etc., see e.g., [7, 8] for terminology). This includes an in-depth description of the content (including where it is coming from and who developed it) [1],” whether [and how] it is tailored to individual circumstances and allows users to track their progress and receive feedback” [6]. This also includes a description of communication delivery channels and – if computer-mediated communication is a component – whether communication was synchronous or asynchronous [6]. It also includes information on presentation strategies [1], including page design principles, average amount of text on pages, presence of hyperlinks to other resources, etc. [1].

	1	2	3	4	5	
subitem not at all important	○	○	○	◉	○	essential

Does your paper address subitem 5-viii? *

Copy and paste relevant sections from the manuscript (include quotes in quotation marks "like this" to indicate direct quotes from your manuscript), or elaborate on this item by providing additional information not in the ms, or briefly explain why the item is not applicable/relevant for your study

The mode of delivery was by a social robot (see 5-vi)

5-ix) Describe use parameters

Describe use parameters (e.g., intended “doses” and optimal timing for use). Clarify what instructions or recommendations were given to the user, e.g., regarding timing, frequency, heaviness of use, if any, or was the intervention used ad libitum.

	1	2	3	4	5	
subitem not at all important	◉	○	○	○	○	essential

Does your paper address subitem 5-ix?

Copy and paste relevant sections from the manuscript (include quotes in quotation marks "like this" to indicate direct quotes from your manuscript), or elaborate on this item by providing additional information not in the ms, or briefly explain why the item is not applicable/relevant for your study

Not applicable.

5-x) Clarify the level of human involvement

Clarify the level of human involvement (care providers or health professionals, also technical assistance) in the e-intervention or as co-intervention (detail number and expertise of professionals involved, if any, as well as “type of assistance offered, the timing and frequency of the support, how it is initiated, and the medium by which the assistance is delivered”. It may be necessary to distinguish between the level of human involvement required for the trial, and the level of human involvement required for a routine application outside of a RCT setting (discuss under item 21 – generalizability).

	1	2	3	4	5	
subitem not at all important	○	○	○	○	◉	essential

Does your paper address subitem 5-x?

Copy and paste relevant sections from the manuscript (include quotes in quotation marks "like this" to indicate direct quotes from your manuscript), or elaborate on this item by providing additional information not in the ms, or briefly explain why the item is not applicable/relevant for your study

The social robot conducted the interview autonomously.

5-xi) Report any prompts/reminders used

Report any prompts/reminders used: Clarify if there were prompts (letters, emails, phone calls, SMS) to use the application, what triggered them, frequency etc. It may be necessary to distinguish between the level of prompts/reminders required for the trial, and the level of prompts/reminders for a routine application outside of a RCT setting (discuss under item 21 – generalizability).

	1	2	3	4	5	
subitem not at all important	(•)	( )	( )	( )	( )	essential

Does your paper address subitem 5-xi? *

Copy and paste relevant sections from the manuscript (include quotes in quotation marks "like this" to indicate direct quotes from your manuscript), or elaborate on this item by providing additional information not in the ms, or briefly explain why the item is not applicable/relevant for your study

Not applicable, the social robot was used at the outpatient clinic once per patient.

5-xii) Describe any co-interventions (incl. training/support)

Describe any co-interventions (incl. training/support): Clearly state any interventions that are provided in addition to the targeted eHealth intervention, as ehealth intervention may not be designed as stand-alone intervention. This includes training sessions and support [1]. It may be necessary to distinguish between the level of training required for the trial, and the level of training for a routine application outside of a RCT setting (discuss under item 21 – generalizability.

	1	2	3	4	5	
subitem not at all important	(•)	( )	( )	( )	( )	essential

Does your paper address subitem 5-xii? *

Copy and paste relevant sections from the manuscript (include quotes in quotation marks "like this" to indicate direct quotes from your manuscript), or elaborate on this item by providing additional information not in the ms, or briefly explain why the item is not applicable/relevant for your study

Not applicable, the social robot conducted the interview autonomously.

6a) Completely defined pre-specified primary and secondary outcome measures, including how and when they were assessed

Does your paper address CONSORT subitem 6a? *

Copy and paste relevant sections from the manuscript (include quotes in quotation marks "like this" to indicate direct quotes from your manuscript), or elaborate on this item by providing additional information not in the ms, or briefly explain why the item is not applicable/relevant for your study

Primary outcome
The most relevant part of a general medical CQI was selected as primary outcome measure[24]. The Dutch CQI has different versions for different specialties. Because not all of the list items were applicable to our study and in the attempt to minimise the burden to the patients, the 10 most relevant questions were selected in advance (Appendix D, Table 2). This selection was done in line with recommendations for shortening the CQI questionnaires[17,25].
Answers to the CQI were scored categorically, ranging from 'no, not at all', 'a little', 'largely', to 'yes, completely'. The granularity of this scale was small, however, and pilot evaluations revealed ceiling effects and skewed distributions. The patients were therefore asked to assign scores on a scale from 1 to 10, with references to these categories (Figure 1).

Did you feel welcome at the outpatient clinic? (Please encircle the number)
No, not at all A little Largely Yes, completely
1 2 3 4 5 6 7 8 9 10
Figure 1 – As an example, one of the 10 CQI questions is presented in its 10-point scale version
The opinion of the informal caregiver accompanying the patient was also recorded using the same questions, albeit reformulated for the informal caregiver's perspective. The answers to each CQI question were averaged across all patients and caregivers in each group. The primary outcome was then calculated as the mean sum of the individual question outcomes.
Secondary outcome
The time duration of the TOPICS-SF-interview was registered as a secondary outcome by observers who witnessed each interview. These observers further used an observation form to record, for each question, the extent to which the patient and caregiver exchanged information on the TOPICS-SF answers (Appendix E, Figure 2). Other potentially relevant events were also recorded (e.g. patient remarks on the interaction). The observers were instructed not to intervene at all. Given that such self-recording of secondary outcomes could not be blinded, observation bias was limited by using alternating trained observers.
The general medical situation of the patient group was categorised according to the mean Frailty Index as follows: 'robust' ($FI \leq 0.095$), 'pre-frail' ($0.095 < FI < 0.20$) and 'frail' ($FI \geq 0.2$) [26]. The total number of reported morbidities per patient was counted, theoretically resulting in a value between 0 and 18.
In the intervention group, four questions based on the Almere model[27] were asked to evaluate the usability of the robot (Appendix F, Table 3). This made it possible to compare these results to our previous work[28,29]. To limit patient burden, survey questions were restricted to three variables: 'Perceived Ease of Use' (2 items), 'Perceived Enjoyment' and 'Trust'[27].

6a-i) Online questionnaires: describe if they were validated for online use and apply CHERRIES items to describe how the questionnaires were designed/deployed

If outcomes were obtained through online questionnaires, describe if they were validated for online use and apply CHERRIES items to describe how the questionnaires were designed/deployed [9].

	1	2	3	4	5	
subitem not at all important	(•)	( )	( )	( )	( )	essential

Does your paper address subitem 6a-i?

Copy and paste relevant sections from manuscript text

Online questionnaires were not used.

6a-ii) Describe whether and how "use" (including intensity of use/dosage) was defined/measured/monitored

Describe whether and how "use" (including intensity of use/dosage) was defined/measured/monitored (logins, logfile analysis, etc.). Use/adoption metrics are important process outcomes that should be reported in any ehealth trial.

	1	2	3	4	5	
subitem not at all important	(•)	( )	( )	( )	( )	essential

Does your paper address subitem 6a-ii?

Copy and paste relevant sections from manuscript text

Intervention was done only once.

6a-iii) Describe whether, how, and when qualitative feedback from participants was obtained

Describe whether, how, and when qualitative feedback from participants was obtained (e.g., through emails, feedback forms, interviews, focus groups).

	1	2	3	4	5	
subitem not at all important	○	○	○	○	◉	essential

Does your paper address subitem 6a-iii?

Copy and paste relevant sections from manuscript text

By Consumer Quality Index (the primary outcome)

6b) Any changes to trial outcomes after the trial commenced, with reasons

Does your paper address CONSORT subitem 6b? *

Copy and paste relevant sections from the manuscript (include quotes in quotation marks "like this" to indicate direct quotes from your manuscript), or elaborate on this item by providing additional information not in the ms, or briefly explain why the item is not applicable/relevant for your study

Not applicable

7a) How sample size was determined

NPT: When applicable, details of whether and how the clustering by care provides or centers was addressed

7a-i) Describe whether and how expected attrition was taken into account when calculating the sample size

Describe whether and how expected attrition was taken into account when calculating the sample size.

	1	2	3	4	5	
subitem not at all important	○	○	○	○	◉	essential

Does your paper address subitem 7a-i?

Copy and paste relevant sections from manuscript title (include quotes in quotation marks "like this" to indicate direct quotes from your manuscript), or elaborate on this item by providing additional information not in the ms, or briefly explain why the item is not applicable/relevant for your study

In our two previous robot studies (conducted with 30 and 40 community-dwelling older volunteers), we found hardly any difference between the answers given to the robot and those give to the healthcare professional[18, 22]. In the current study, therefore, we focused on the quality of care perceived by patients and caregivers, hypothesising that the robot interview would also not be valued less by the intervention group. For this reason, a non-inferiority sample-size calculation was applied, specifying that the mean CQI of the intervention group should not be lower than the mean CQI of the control group minus 1.0, with a standard deviation of 1.5, α = .05 and power = 1-β = .90[25,30]. The difference value of 1.0 is based on the guideline proposed by Ringash et al., which defines 10% of the PROM scale range as a meaningful difference[31]. This calculation resulted in a sample size (n) of 39 patients per group (78 in total).

7b) When applicable, explanation of any interim analyses and stopping guidelines

Does your paper address CONSORT subitem 7b? *

Copy and paste relevant sections from the manuscript (include quotes in quotation marks "like this" to indicate direct quotes from your manuscript), or elaborate on this item by providing additional information not in the ms, or briefly explain why the item is not applicable/relevant for your study

Not applicable, no interim analyses were conducted.

8a) Method used to generate the random allocation sequence

NPT: When applicable, how care providers were allocated to each trial group

Does your paper address CONSORT subitem 8a? *

Copy and paste relevant sections from the manuscript (include quotes in quotation marks "like this" to indicate direct quotes from your manuscript), or elaborate on this item by providing additional information not in the ms, or briefly explain why the item is not applicable/relevant for your study

Patients were randomised using a computer-generated list.

8b) Type of randomisation; details of any restriction (such as blocking and block size)

Does your paper address CONSORT subitem 8b? *

Copy and paste relevant sections from the manuscript (include quotes in quotation marks "like this" to indicate direct quotes from your manuscript), or elaborate on this item by providing additional information not in the ms, or briefly explain why the item is not applicable/relevant for your study

Patients were randomised using a computer-generated list and assigned to either the intervention or the control group, in sequence of admission.

9) Mechanism used to implement the random allocation sequence (such as sequentially numbered containers), describing any steps taken to conceal the sequence until interventions were assigned

Does your paper address CONSORT subitem 9? *

Copy and paste relevant sections from the manuscript (include quotes in quotation marks "like this" to indicate direct quotes from your manuscript), or elaborate on this item by providing additional information not in the ms, or briefly explain why the item is not applicable/relevant for your study

Patients were assigned to either the intervention or the control group, in sequence of admission, using the computer-generated list.

10) Who generated the random allocation sequence, who enrolled participants, and who assigned participants to interventions

Does your paper address CONSORT subitem 10? *

Copy and paste relevant sections from the manuscript (include quotes in quotation marks "like this" to indicate direct quotes from your manuscript), or elaborate on this item by providing additional information not in the ms, or briefly explain why the item is not applicable/relevant for your study

The random allocation sequence list was computer-generated. Patients were (after inclusion, see before) assigned to either the intervention or the control group, in sequence of admission.

11a) If done, who was blinded after assignment to interventions (for example, participants, care providers, those assessing outcomes) and how

NPT: Whether or not administering co-interventions were blinded to group assignment

11a-i) Specify who was blinded, and who wasn’t

Specify who was blinded, and who wasn’t. Usually, in web-based trials it is not possible to blind the participants [1, 3] (this should be clearly acknowledged), but it may be possible to blind outcome assessors, those doing data analysis or those administering co-interventions (if any).

	1	2	3	4	5	
subitem not at all important	◉	○	○	○	○	essential

Does your paper address subitem 11a-i? *

Copy and paste relevant sections from the manuscript (include quotes in quotation marks "like this" to indicate direct quotes from your manuscript), or elaborate on this item by providing additional information not in the ms, or briefly explain why the item is not applicable/relevant for your study

The nature of the intervention prevented the blinding of group allocation, and data acquisition could not be blinded from the patient perspective, given that the data were self-reported.

11a-ii) Discuss e.g., whether participants knew which intervention was the “intervention of interest” and which one was the “comparator”

Informed consent procedures (4a-ii) can create biases and certain expectations - discuss e.g., whether participants knew which intervention was the “intervention of interest” and which one was the “comparator”.

	1	2	3	4	5	
subitem not at all important	○	○	○	○	◉	essential

Does your paper address subitem 11a-ii?

Copy and paste relevant sections from the manuscript (include quotes in quotation marks "like this" to indicate direct quotes from your manuscript), or elaborate on this item by providing additional information not in the ms, or briefly explain why the item is not applicable/relevant for your study

Yes they knew: The nature of the intervention prevented the blinding of group allocation, and data acquisition could not be blinded from the patient perspective, given that the data were self-reported.

11b) If relevant, description of the similarity of interventions

(this item is usually not relevant for ehealth trials as it refers to similarity of a placebo or sham intervention to a active medication/intervention)

Does your paper address CONSORT subitem 11b? *

Copy and paste relevant sections from the manuscript (include quotes in quotation marks "like this" to indicate direct quotes from your manuscript), or elaborate on this item by providing additional information not in the ms, or briefly explain why the item is not applicable/relevant for your study

In the intervention pathway, the healthcare professional started the interview with several open-ended questions on the patient’s general health status. This was followed by the introduction of the robot, which subsequently conducted the TOPICS-SF questionnaire interview. Upon completing the interview, the robot generated a report of the PROM and Frailty Index results, including the (Instrumental) Activities of Daily Living scores. This report was input for subsequent interactions between the patient and the healthcare professional within the context of shared decision-making[21]. The robot-patient interaction is detailed in Appendix C.

In the control group, following the initial general talk, the healthcare professional started the structured TOPICS-SF questionnaire. The results were discussed with the patient, and the other parts of the medical examination and management plan were carried out.

12a) Statistical methods used to compare groups for primary and secondary outcomes

NPT: When applicable, details of whether and how the clustering by care providers or centers was addressed

Does your paper address CONSORT subitem 12a? *

Copy and paste relevant sections from the manuscript (include quotes in quotation marks "like this" to indicate direct quotes from your manuscript), or elaborate on this item by providing additional information not in the ms, or briefly explain why the item is not applicable/relevant for your study

Data were stored in Castor, a cloud-based medical data management system (Castor, Amsterdam, Netherlands). Intention-to-treat analysis was performed using SPSS statistical software (version 25; IBM, Armonk, NY, USA) and Microsoft Excel (Office365; Microsoft, Redmond, WA, USA). Because not all data were reported by patients or caregivers, the number of patients to which variables relate are reported separately. Missing values were not considered random, and thus not imputed. Normally distributed values are presented as means, with standard deviations in parentheses. Because the target sample size (n) was larger than 25, we applied the central limit theorem and assumed normality on the part of the summed score for the CQI questionnaire. Groups were compared using independent samples t-tests and, in case of non-normality, the Mann-Whitney U test. For significant effects or effect trends, effect sizes were calculated as Cohen's d.

12a-i) Imputation techniques to deal with attrition / missing values

Imputation techniques to deal with attrition / missing values: Not all participants will use the intervention/comparator as intended and attrition is typically high in ehealth trials. Specify how participants who did not use the application or dropped out from the trial were treated in the statistical analysis (a complete case analysis is strongly discouraged, and simple imputation techniques such as LOCF may also be problematic [4]).

	1	2	3	4	5	
subitem not at all important	◉	○	○	○	○	essential

Does your paper address subitem 12a-i? *

Copy and paste relevant sections from the manuscript (include quotes in quotation marks "like this" to indicate direct quotes from your manuscript), or elaborate on this item by providing additional information not in the ms, or briefly explain why the item is not applicable/relevant for your study

Missing values were not considered random, and thus not imputed.

12b) Methods for additional analyses, such as subgroup analyses and adjusted analyses

Does your paper address CONSORT subitem 12b? *

Copy and paste relevant sections from the manuscript (include quotes in quotation marks "like this" to indicate direct quotes from your manuscript), or elaborate on this item by providing additional information not in the ms, or briefly explain why the item is not applicable/relevant for your study

Not applicable

X26) REB/IRB Approval and Ethical Considerations [recommended as subheading under "Methods"] (not a CONSORT item)

X26-i) Comment on ethics committee approval

	1	2	3	4	5	
subitem not at all important	○	○	○	○	◉	essential

Does your paper address subitem X26-i?

Copy and paste relevant sections from the manuscript (include quotes in quotation marks "like this" to indicate direct quotes from your manuscript), or elaborate on this item by providing additional information not in the ms, or briefly explain why the item is not applicable/relevant for your study

The study protocol was approved by the Institutional Review Boards of both hospitals.

x26-ii) Outline informed consent procedures

Outline informed consent procedures e.g., if consent was obtained offline or online (how? Checkbox, etc.?), and what information was provided (see 4a-ii). See [6] for some items to be included in informed consent documents.

	1	2	3	4	5	
subitem not at all important	○	○	○	○	◉	essential

Does your paper address subitem X26-ii?

Copy and paste relevant sections from the manuscript (include quotes in quotation marks "like this" to indicate direct quotes from your manuscript), or elaborate on this item by providing additional information not in the ms, or briefly explain why the item is not applicable/relevant for your study

All patients granted written informed consent. A specific form was used to obtain consent.

X26-iii) Safety and security procedures

Safety and security procedures, incl. privacy considerations, and any steps taken to reduce the likelihood or detection of harm (e.g., education and training, availability of a hotline)

	1	2	3	4	5	
subitem not at all important	○	○	○	○	◉	essential

Does your paper address subitem X26-iii?

Copy and paste relevant sections from the manuscript (include quotes in quotation marks "like this" to indicate direct quotes from your manuscript), or elaborate on this item by providing additional information not in the ms, or briefly explain why the item is not applicable/relevant for your study

The study was conducted according to the principles of the Declaration of Helsinki (2013), in accordance with the Medical Research Involving Human Subjects Act (WMO) and the CONSORT guidelines for Randomised Controlled Trials, including the extension for non-inferiority trials[32].

RESULTS

13a) For each group, the numbers of participants who were randomly assigned, received intended treatment, and were analysed for the primary outcome

NPT: The number of care providers or centers performing the intervention in each group and the number of patients treated by each care provider in each center

Does your paper address CONSORT subitem 13a? *

Copy and paste relevant sections from the manuscript (include quotes in quotation marks "like this" to indicate direct quotes from your manuscript), or elaborate on this item by providing additional information not in the ms, or briefly explain why the item is not applicable/relevant for your study

A patient flowchart is included. In summary, 110 patients were invited, 80 were randomised: 40 in intervention group and 40 in control group.

13b) For each group, losses and exclusions after randomisation, together with reasons

Does your paper address CONSORT subitem 13b? (NOTE: Preferably, this is shown in a CONSORT flow diagram) *

Copy and paste relevant sections from the manuscript (include quotes in quotation marks "like this" to indicate direct quotes from your manuscript), or elaborate on this item by providing additional information not in the ms, or briefly explain why the item is not applicable/relevant for your study

Two patients dropped out during the experiment after randomisation, one patient turned out to have cognitive problems that made it impossible to complete the robot interaction, and one patient chose to discontinue the interview with the robot after nine questions because ‘she did not like the robot’. Two CQI measurements failed because patients refused the rating.

13b-i) Attrition diagram

Strongly recommended: An attrition diagram (e.g., proportion of participants still logging in or using the intervention/comparator in each group plotted over time, similar to a survival curve) or other figures or tables demonstrating usage/dose/engagement.

	1	2	3	4	5	
subitem not at all important	○	○	○	○	◉	essential

Does your paper address subitem 13b-i?

Copy and paste relevant sections from the manuscript or cite the figure number if applicable (include quotes in quotation marks "like this" to indicate direct quotes from your manuscript), or elaborate on this item by providing additional information not in the ms, or briefly explain why the item is not applicable/relevant for your study

See figure 2 in the manuscript.

14a) Dates defining the periods of recruitment and follow-up

Does your paper address CONSORT subitem 14a? *

Copy and paste relevant sections from the manuscript (include quotes in quotation marks "like this" to indicate direct quotes from your manuscript), or elaborate on this item by providing additional information not in the ms, or briefly explain why the item is not applicable/relevant for your study

The setting concerned two outpatient clinics in the period July–December 2019.

14a-i) Indicate if critical “secular events” fell into the study period

Indicate if critical “secular events” fell into the study period, e.g., significant changes in Internet resources available or “changes in computer hardware or Internet delivery resources”

	1	2	3	4	5	
subitem not at all important	◉	○	○	○	○	essential

Does your paper address subitem 14a-i?

Copy and paste relevant sections from the manuscript (include quotes in quotation marks "like this" to indicate direct quotes from your manuscript), or elaborate on this item by providing additional information not in the ms, or briefly explain why the item is not applicable/relevant for your study

Not applicable.

14b) Why the trial ended or was stopped (early)

Does your paper address CONSORT subitem 14b? *

Copy and paste relevant sections from the manuscript (include quotes in quotation marks "like this" to indicate direct quotes from your manuscript), or elaborate on this item by providing additional information not in the ms, or briefly explain why the item is not applicable/relevant for your study

Inclusion was stopped upon reaching 80 included patients (38 female, 42 male, mean age M=77.8 years old, SD=7.3, range 60−91).

15) A table showing baseline demographic and clinical characteristics for each group

NPT: When applicable, a description of care providers (case volume, qualification, expertise, etc.) and centers (volume) in each group

Does your paper address CONSORT subitem 15? *

Copy and paste relevant sections from the manuscript (include quotes in quotation marks "like this" to indicate direct quotes from your manuscript), or elaborate on this item by providing additional information not in the ms, or briefly explain why the item is not applicable/relevant for your study

Yes. Since these data are in Figure 2 and Table 1, we have to refer to the manuscript.

15-i) Report demographics associated with digital divide issues

In ehealth trials it is particularly important to report demographics associated with digital divide issues, such as age, education, gender, social-economic status, computer/Internet/ehealth literacy of the participants, if known.

	1	2	3	4	5	
subitem not at all important	○	○	○	○	◉	essential

Does your paper address subitem 15-i? *

Copy and paste relevant sections from the manuscript (include quotes in quotation marks "like this" to indicate direct quotes from your manuscript), or elaborate on this item by providing additional information not in the ms, or briefly explain why the item is not applicable/relevant for your study

Please refer to Table 1 in the manuscript and Appendix G.

16) For each group, number of participants (denominator) included in each analysis and whether the analysis was by original assigned groups

16-i) Report multiple “denominators” and provide definitions

Report multiple “denominators” and provide definitions: Report N’s (and effect sizes) “across a range of study participation [and use] thresholds” [1], e.g., N exposed, N consented, N used more than x times, N used more than y weeks, N participants “used” the intervention/comparator at specific pre-defined time points of interest (in absolute and relative numbers per group). Always clearly define “use” of the intervention.

	1	2	3	4	5	
subitem not at all important	○	○	○	○	◉	essential

Does your paper address subitem 16-i? *

Copy and paste relevant sections from the manuscript (include quotes in quotation marks "like this" to indicate direct quotes from your manuscript), or elaborate on this item by providing additional information not in the ms, or briefly explain why the item is not applicable/relevant for your study

The Frailty Index (FI) for the group as a whole ranged from 0.07 to 0.68 (M=0.26, SD=0.15). The mean FI for the control group (M=0.26, SD=0.15) and the intervention group (M=0.25, SD=0.15) were similar (P=.85). Four patients could be categorised as robust, 32 as pre-frail and 37 as frail, and 23 patients (29%) had been diagnosed with dementia. The average number of morbidities per patient was 3.9 (SD=2.7, n=75). Main patient baseline clinical data for each group are included in Table 1; extended data are provided in Appendix G.
The total CQI scores recorded for patients and caregivers are presented graphically in Figure 3. There was no significant difference in the total patient CQI score for the intervention group (M=9.19, SD=0.83, n=38) and the control group (M=9.00, SD=0.70, n=38); t(74)=1.07, P=.29, 95% CI (-0.16 to 0.54). There also was no significant difference in the total informal caregiver CQI score for the intervention group (M=9.15, SD=0.78, n=32) and the control group (M=9.12, SD=0.61, n=36); t(66)=0.19, P=.85, 95% CI (-0.31 to 0.37). The CQI score for each question is included in Appendix H, and the total CQI distribution is presented in Appendix I.
Within the pathways, the mean duration of taking the TOPICS-SF with the robot was 17.6 minutes (SD=5.6, n=39), as compared to 15.1 minutes in the control group (SD=10.8, n=38). The difference was not significant: t(75)=1.27, P=.21, 95% CI (-1.40 to 6.37). It should be noted that observations showed that healthcare professionals regularly skipped questions.
For the intervention group only, the mean scores for Perceived Enjoyment, Perceived Ease of Use (2 items) and Trust with regard to the robot interaction were recorded (Appendix J). There was no significant difference in Perceived Enjoyment between patients (Mpat=7.62, SDpat=2.28) and caregivers (Mcg=7.29, SDcg=2.36), t(59)=0.55, P=.59, 95% CI (-0.87 to 1.53). There was also no significant difference in perceived Ease of Use in terms of having sufficient time between patients (Mpat=8.42, SDpat=1.71) and caregivers (Mcg=8.33, SDcg=1.15), t(58)=0.21, P=.84, 95% CI (-0.71 to 0.88), nor was there a significant difference in Perceived Ease of Use in terms of easy answering between patients (Mpat=8.03, SDpat=1.94) and caregivers (Mcg=7.58, SDcg=2.02), t(59)=0.86, p=0.39, 95% CI (-0.58 to 1.47). Trust scores were higher for patients (Mpat=8.22, SDpat=1.84) than for caregivers (Mcg=7.08, SDcg=2.47), t(58)=2.07, P=.04, 95% CI (0.04 to 2.24), Cohen's d=0.55. Of the 36 caregivers in the intervention group who answered the CQI questions, only 24 also answered these questions on robot appreciation. The caregivers who did not answer argued that it was better for the patients to answer themselves, as they had been the ones to talk to the robot.

16-ii) Primary analysis should be intent-to-treat

Primary analysis should be intent-to-treat, secondary analyses could include comparing only “users”, with the appropriate caveats that this is no longer a randomized sample (see 18-i).

	1	2	3	4	5	
subitem not at all important	○	◉	○	○	○	essential

Does your paper address subitem 16-ii?

Copy and paste relevant sections from the manuscript (include quotes in quotation marks "like this" to indicate direct quotes from your manuscript), or elaborate on this item by providing additional information not in the ms, or briefly explain why the item is not applicable/relevant for your study

The primary analysis was intent-to-treat.

17a) For each primary and secondary outcome, results for each group, and the estimated effect size and its precision (such as 95% confidence interval)

Does your paper address CONSORT subitem 17a? *

Copy and paste relevant sections from the manuscript (include quotes in quotation marks "like this" to indicate direct quotes from your manuscript), or elaborate on this item by providing additional information not in the ms, or briefly explain why the item is not applicable/relevant for your study

See answer to 16-i.

17a-i) Presentation of process outcomes such as metrics of use and intensity of use

In addition to primary/secondary (clinical) outcomes, the presentation of process outcomes such as metrics of use and intensity of use (dose, exposure) and their operational definitions is critical. This does not only refer to metrics of attrition (13-b) (often a binary variable), but also to more continuous exposure metrics such as “average session length”. These must be accompanied by a technical description how a metric like a “session” is defined (e.g., timeout after idle time) [1] (report under item 6a).

	1	2	3	4	5	
subitem not at all important	○	○	○	○	◉	essential

Does your paper address subitem 17a-i?

Copy and paste relevant sections from the manuscript (include quotes in quotation marks "like this" to indicate direct quotes from your manuscript), or elaborate on this item by providing additional information not in the ms, or briefly explain why the item is not applicable/relevant for your study

See answer to 16-i

17b) For binary outcomes, presentation of both absolute and relative effect sizes is recommended

Does your paper address CONSORT subitem 17b? *

Copy and paste relevant sections from the manuscript (include quotes in quotation marks "like this" to indicate direct quotes from your manuscript), or elaborate on this item by providing additional information not in the ms, or briefly explain why the item is not applicable/relevant for your study

No binary outcomes were recorded.

18) Results of any other analyses performed, including subgroup analyses and adjusted analyses, distinguishing pre-specified from exploratory

Does your paper address CONSORT subitem 18? *

Copy and paste relevant sections from the manuscript (include quotes in quotation marks "like this" to indicate direct quotes from your manuscript), or elaborate on this item by providing additional information not in the ms, or briefly explain why the item is not applicable/relevant for your study

Patients and caregivers did not discuss the TOPICS-SF answer options any more during the interviews with the robot (M=3.5, SD=4.0, n=35, range 0-16) than was the case in the control group (M=2.9, SD=2.5, n=23, range 0-8), t(56)=0.751, P=.46, 95% CI (-1.17 to 2,57). It was further observed that, at the start of the interview, patients sometimes answered before the robot was finished speaking. This well-known barge-in effect occurred despite the fact that the robot had instructed patients to wait for the blue bar to appear at the top of the tablet before speaking[33]. Most patients learned after three or four questions that it was better to wait a short while before answering, as they would otherwise have to repeat their answers. Informal caregivers occasionally helped the patients when necessary. For example, because one patient spoke a local Dutch dialect that was not understood by the robot, the patient's caregiver answered instead.

18-i) Subgroup analysis of comparing only users

A subgroup analysis of comparing only users is not uncommon in ehealth trials, but if done, it must be stressed that this is a self-selected sample and no longer an unbiased sample from a randomized trial (see 16-iii).

	1	2	3	4	5	
subitem not at all important	○	○	○	◉	○	essential

Does your paper address subitem 18-i?

Copy and paste relevant sections from the manuscript (include quotes in quotation marks "like this" to indicate direct quotes from your manuscript), or elaborate on this item by providing additional information not in the ms, or briefly explain why the item is not applicable/relevant for your study

For the intervention group only, the mean scores for Perceived Enjoyment, Perceived Ease of Use (2 items) and Trust with regard to the robot interaction were recorded (Appendix J). There was no significant difference in Perceived Enjoyment between patients (Mpat=7.62, SDpat=2.28) and caregivers (Mcg=7.29, SDcg=2.36), t(59)=0.55, P=.59, 95% CI (-0.87 to 1.53). There was also no significant difference in perceived Ease of Use in terms of having sufficient time between patients (Mpat=8.42, SDpat=1.71) and caregivers (Mcg=8.33, SDcg=1.15), t(58)=0.21, P=.84, 95% CI (-0.71 to 0.88), nor was there a significant difference in Perceived Ease of Use in terms of easy answering between patients (Mpat=8.03, SDpat=1.94) and caregivers (Mcg=7.58, SDcg=2.02), t(59)=0.86, p=0.39, 95% CI (-0.58 to 1.47). Trust scores were higher for patients (Mpat=8.22, SDpat=1.84) than for caregivers (Mcg=7.08, SDcg=2.47), t(58)=2.07, P=.04, 95% CI (0.04 to 2.24), Cohen's d=0.55. Of the 36 caregivers in the intervention group who answered the CQI questions, only 24 also answered these questions on robot appreciation. The caregivers who did not answer argued that it was better for the patients to answer themselves, as they had been the ones to talk to the robot.

19) All important harms or unintended effects in each group

(for specific guidance see CONSORT for harms)

Does your paper address CONSORT subitem 19? *

Copy and paste relevant sections from the manuscript (include quotes in quotation marks "like this" to indicate direct quotes from your manuscript), or elaborate on this item by providing additional information not in the ms, or briefly explain why the item is not applicable/relevant for your study

No important incidents of harm or unintended effects were observed or reported.

19-i) Include privacy breaches, technical problems

Include privacy breaches, technical problems. This does not only include physical “harm” to participants, but also incidents such as perceived or real privacy breaches [1], technical problems, and other unexpected/unintended incidents. “Unintended effects” also includes unintended positive effects [2].

	1	2	3	4	5	
subitem not at all important	(selected)					essential

Does your paper address subitem 19-i?

Copy and paste relevant sections from the manuscript (include quotes in quotation marks "like this" to indicate direct quotes from your manuscript), or elaborate on this item by providing additional information not in the ms, or briefly explain why the item is not applicable/relevant for your study

No important incidents of harm or unintended effects were observed or reported.

19-ii) Include qualitative feedback from participants or observations from staff/researchers

Include qualitative feedback from participants or observations from staff/researchers, if available, on strengths and shortcomings of the application, especially if they point to unintended/unexpected effects or uses. This includes (if available) reasons for why people did or did not use the application as intended by the developers.

	1	2	3	4	5	
subitem not at all important				(selected)		essential

Does your paper address subitem 19-ii?

Copy and paste relevant sections from the manuscript (include quotes in quotation marks "like this" to indicate direct quotes from your manuscript), or elaborate on this item by providing additional information not in the ms, or briefly explain why the item is not applicable/relevant for your study

Observations
Patients and caregivers did not discuss the TOPICS-SF answer options any more during the interviews with the robot (M=3.5, SD=4.0, n=35, range 0-16) than was the case in the control group (M=2.9, SD=2.5, n=23, range 0-8), t(56)=0.751, P=.46, 95% CI (-1.17 to 2,57). It was further observed that, at the start of the interview, patients sometimes answered before the robot was finished speaking. This well-known barge-in effect occurred despite the fact that the robot had instructed patients to wait for the blue bar to appear at the top of the tablet before speaking[33]. Most patients learned after three or four questions that it was better to wait a short while before answering, as they would otherwise have to repeat their answers. Informal caregivers occasionally helped the patients when necessary. For example, because one patient spoke a local Dutch dialect that was not understood by the robot, the patient's caregiver answered instead.

DISCUSSION

22) Interpretation consistent with results, balancing benefits and harms, and considering other relevant evidence

NPT: In addition, take into account the choice of the comparator, lack of or partial blinding, and unequal expertise of care providers or centers in each group

22-i) Restate study questions and summarize the answers suggested by the data, starting with primary outcomes and process outcomes (use)

Restate study questions and summarize the answers suggested by the data, starting with primary outcomes and process outcomes (use).

	1	2	3	4	5	
subitem not at all important	○	○	○	○	◉	essential

Does your paper address subitem 22-i? *

Copy and paste relevant sections from the manuscript (include quotes in quotation marks "like this" to indicate direct quotes from your manuscript), or elaborate on this item by providing additional information not in the ms, or briefly explain why the item is not applicable/relevant for your study

We found that the perceptions of older patients and caregivers concerning quality of care were no different from the perceptions of quality of care in a pathway in which all interactions were carried out by healthcare professionals. This confirmed our hypothesis of non-inferiority.

22-ii) Highlight unanswered new questions, suggest future research

Highlight unanswered new questions, suggest future research.

	1	2	3	4	5	
subitem not at all important	○	○	○	◉	○	essential

Does your paper address subitem 22-ii?

Copy and paste relevant sections from the manuscript (include quotes in quotation marks "like this" to indicate direct quotes from your manuscript), or elaborate on this item by providing additional information not in the ms, or briefly explain why the item is not applicable/relevant for your study

In the course of our study, we learned that one important further step in improving robot technology involves developing the ability to speak and listen at the same time, thus allowing for ‘barging-in’ by patients. Although such technology does exist, it was not implemented in the robot used in this study. Moreover, the quality of the robot’s speech recognition depended on its focus on the interlocutor, which was controlled by the built-in ‘human engagement’ function. Improving the controllability of this function in terms of both speech and body motions would help to build rapport with users.

20) Trial limitations, addressing sources of potential bias, imprecision, and, if relevant, multiplicity of analyses

20-i) Typical limitations in ehealth trials

Typical limitations in ehealth trials: Participants in ehealth trials are rarely blinded. Ehealth trials often look at a multiplicity of outcomes, increasing risk for a Type I error. Discuss biases due to non-use of the intervention/usability issues, biases through informed consent procedures, unexpected events.

	1	2	3	4	5	
subitem not at all important	○	○	○	●	○	essential

Does your paper address subitem 20-i? *

Copy and paste relevant sections from the manuscript (include quotes in quotation marks "like this" to indicate direct quotes from your manuscript), or elaborate on this item by providing additional information not in the ms, or briefly explain why the item is not applicable/relevant for your study

Strengths and weaknesses of the study
The major strength of this study is that it is the first multi-centre randomised controlled trial on the acquisition of routine collected PROM data with a social robot among older adult patients within an integrated care pathway. The non-inferiority results of this trial suggest that an adequately designed social robot could be acceptable for use with older adult patients and their informal caregivers as part of an integrated care pathway, under the indirect supervision of a healthcare professional.
Despite this strength, this study is also subject to several limitations. First, it was not possible to blind the assignment of patients to groups. Second, the between-subject design did not allow any comparative-accuracy analyses of the answers. In our previous study, however, the results indicated moderate to good agreement between scores with and without robot[29].
Strength and weaknesses of the study in relation to other studies
The results confirm and extend those of previous studies on the use of robots outside the hospital context[9,11,14,15]. For example, Olde Keizer et al. concluded that social robots could potentially monitor and train the health of frail older adults, but they also identified some critical usability challenges[35].

21) Generalisability (external validity, applicability) of the trial findings

NPT: External validity of the trial findings according to the intervention, comparators, patients, and care providers or centers involved in the trial

21-i) Generalizability to other populations

Generalizability to other populations: In particular, discuss generalizability to a general Internet population, outside of a RCT setting, and general patient population, including applicability of the study results for other organizations

	1	2	3	4	5	
subitem not at all important	○	○	○	○	◉	essential

Does your paper address subitem 21-i?

Copy and paste relevant sections from the manuscript (include quotes in quotation marks "like this" to indicate direct quotes from your manuscript), or elaborate on this item by providing additional information not in the ms, or briefly explain why the item is not applicable/relevant for your study

In terms of generalisability, the patient group in this study was more frail and had more substantial multimorbidity than is the case for the general hospital population. Communication with the robot could possibly be even easier for the general hospital population. For this reason, and because the TOPICS-SF questionnaire is similar to many available PROMs, it is plausible that the results can be generalised to most adults admitted to hospitals, as well as to most care pathways. The results thus suggest that robot assistance could be implemented more broader without affecting perceived quality of care.

21-ii) Discuss if there were elements in the RCT that would be different in a routine application setting

Discuss if there were elements in the RCT that would be different in a routine application setting (e.g., prompts/reminders, more human involvement, training sessions or other co-interventions) and what impact the omission of these elements could have on use, adoption, or outcomes if the intervention is applied outside of a RCT setting.

	1	2	3	4	5	
subitem not at all important	○	○	○	◉	○	essential

Does your paper address subitem 21-ii?

Copy and paste relevant sections from the manuscript (include quotes in quotation marks "like this" to indicate direct quotes from your manuscript), or elaborate on this item by providing additional information not in the ms, or briefly explain why the item is not applicable/relevant for your study

The observations and experiences gained in this experiment could also be translated into a number of clinical recommendations. First, the introduction of a social robot should lead to a carefully prepared rearrangement of tasks amongst the healthcare professionals within a pathway of care. Second, for reasons of patient privacy and the intelligibility of the patient's utterances to the robot, the robot should be a fixed element in an outpatient room. Third, the integration between the robot and the hospital information system should be designed carefully and incorporated into the EHR system.

OTHER INFORMATION

23) Registration number and name of trial registry

Does your paper address CONSORT subitem 23? *

Copy and paste relevant sections from the manuscript (include quotes in quotation marks "like this" to indicate direct quotes from your manuscript), or elaborate on this item by providing additional information not in the ms, or briefly explain why the item is not applicable/relevant for your study

The trial was registered at ClinicalTrials.gov with reference NCT03857789, status completed.

24) Where the full trial protocol can be accessed, if available

Does your paper address CONSORT subitem 24? *

Cite a Multimedia Appendix, other reference, or copy and paste relevant sections from the manuscript (include quotes in quotation marks "like this" to indicate direct quotes from your manuscript), or elaborate on this item by providing additional information not in the ms, or briefly explain why the item is not applicable/relevant for your study

The trial was registered at ClinicalTrials.gov with reference NCT03857789 and the protocol can be found there.

25) Sources of funding and other support (such as supply of drugs), role of funders

Does your paper address CONSORT subitem 25? *

Copy and paste relevant sections from the manuscript (include quotes in quotation marks "like this" to indicate direct quotes from your manuscript), or elaborate on this item by providing additional information not in the ms, or briefly explain why the item is not applicable/relevant for your study

Your answer

This is a required question

X27) Conflicts of Interest (not a CONSORT item)

X27-i) State the relation of the study team towards the system being evaluated

In addition to the usual declaration of interests (financial or otherwise), also state the relation of the study team towards the system being evaluated, i.e., state if the authors/evaluators are distinct from or identical with the developers/sponsors of the intervention.

	1	2	3	4	5	
subitem not at all important	○	○	◉	○	○	essential

Does your paper address subitem X27-i?

Copy and paste relevant sections from the manuscript (include quotes in quotation marks "like this" to indicate direct quotes from your manuscript), or elaborate on this item by providing additional information not in the ms, or briefly explain why the item is not applicable/relevant for your study

This work has been funded as a research partnership project between the Dutch technical universities and the Radboud university medical center.

About the CONSORT EHEALTH checklist

As a result of using this checklist, did you make changes in your manuscript? *

- ( ) yes, major changes
- ( ) yes, minor changes
- (x) no

What were the most important changes you made as a result of using this checklist?

Not applicable.

How much time did you spend on going through the checklist INCLUDING making changes in your manuscript *

90 minutes

As a result of using this checklist, do you think your manuscript has improved? *

- ( ) yes
- (x) no
- ( ) Other:

Would you like to become involved in the CONSORT EHEALTH group?

This would involve for example becoming involved in participating in a workshop and writing an "Explanation and Elaboration" document

- ( ) yes
- (x) no
- ( ) Other:

Any other comments or questions on CONSORT EHEALTH

We used the CONSORT guidelines for noninferiority trials and for the abstracts before completing this form, and I presume that therefore we did not have to make changes to the manuscript.

STOP - Save this form as PDF before you click submit

To generate a record that you filled in this form, we recommend to generate a PDF of this page (on a Mac, simply select "print" and then select "print as PDF") before you submit it.

When you submit your (revised) paper to JMIR, please upload the PDF as supplementary file.

Don't worry if some text in the textboxes is cut off, as we still have the complete information in our database. Thank you!

Final step: Click submit !

Click submit so we have your answers in our database!

Submit

Never submit passwords through Google Forms.

This content is neither created nor endorsed by Google. Report Abuse - Terms of Service - Privacy Policy

Google Forms
